# Mature chromatin packing domains persist after RAD21 depletion in 3D

**DOI:** 10.1126/sciadv.adp0855

**Published:** 2025-01-24

**Authors:** Wing Shun Li, Lucas M. Carter, Luay Matthew Almassalha, Ruyi Gong, Emily M. Pujadas-Liwag, Tiffany Kuo, Kyle L. MacQuarrie, Marcelo Carignano, Cody Dunton, Vinayak Dravid, Masato T. Kanemaki, Igal Szleifer, Vadim Backman

**Affiliations:** ^1^ Applied Physics Program, Northwestern University, Evanston, IL 60208, USA.; ^2^ Department of Biomedical Engineering, Northwestern University, Evanston, IL 60208, USA.; ^3^ Center for Physical Genomics and Engineering, Northwestern University, Evanston, IL 60208, USA.; ^4^ IBIS Interdisciplinary Biological Sciences Graduate Program, Northwestern University, Evanston, IL 60208, USA.; ^5^ Department of Gastroenterology and Hepatology, Northwestern Memorial Hospital, Chicago, IL 60611, USA.; ^6^ Stanley Manne Children’s Research Institute, Ann & Robert H. Lurie Children’s Hospital of Chicago, Chicago, IL 60611, USA.; ^7^ Northwestern University Feinberg School of Medicine, Chicago, IL 60611, USA.; ^8^ Department of Chemistry, Northwestern University, Evanston, IL 60208, USA.; ^9^ Materials Science and Engineering, Northwestern University, Evanston, IL 60208, USA.; ^10^ Northwestern University Atomic and Nanoscale Characterization Experimental (NUANCE) Center, Northwestern University, Evanston, IL 60208, USA.; ^11^ International Institute for Nanotechnology (IIN), Northwestern University, Evanston, IL 60208, USA.; ^12^ Department of Chromosome Science, National Institute of Genetics, Mishima, Shizuoka 411-8540, Japan.; ^13^ Graduate Institute for Advanced Studies, SOKENDAI, Yata 1111, Mishima, Shizuoka 411-8540, Japan.; ^14^ Department of Biological Science, The University of Tokyo, Bunkyo-ku, Tokyo 113-0033, Japan.

## Abstract

Understanding chromatin organization requires integrating measurements of genome connectivity and physical structure. It is well established that cohesin is essential for TAD and loop connectivity features in Hi-C, but the corresponding change in physical structure has not been studied using electron microscopy. Pairing chromatin scanning transmission electron tomography with multiomic analysis and single-molecule localization microscopy, we study the role of cohesin in regulating the conformationally defined chromatin nanoscopic packing domains. Our results indicate that packing domains are not physical manifestation of TADs. Using electron microscopy, we found that only 20% of packing domains are lost upon RAD21 depletion. The effect of RAD21 depletion is restricted to small, poorly packed (nascent) packing domains. In addition, we present evidence that cohesin-mediated loop extrusion generates nascent domains that undergo maturation through nucleosome posttranslational modifications. Our results demonstrate that a 3D genomic structure, composed of packing domains, is generated through cohesin activity and nucleosome modifications.

## Teaser

RAD21 contributes to nascent packing domain formation, but maturation requires nucleosome posttranslational modifications.

## INTRODUCTION

Our understanding of the mechanisms that constrain chromatin organization in four-dimensional (4D) space is crucial to resolving how DNA replication, repair, and RNA transcription are regulated (*1*–*6*). Furthermore, distortion in chromatin organization is associated with multiple disease processes, including numerous malignancies (*1*, *2*, *5*, *7*–*10*). Since the emergence of high-throughput chromatin conformation capture (Hi-C) sequencing, researchers have worked to understand the mechanisms that result in the formation of topologically associated domains (TADs) and how these domains correspond to physical structures within individual cells (*11*–*17*). From their initial description as foci of higher-than expected contact probability in ensemble population measurements, several key features of TADs have been described. TADs are predominantly associated with cohesin-mediated loop extrusion in association with CCCTC-binding factor (CTCF) binding motifs and potentially act as boundary-mediating elements in supranucleosomal chromatin organization (*13*, *15*, *18*–*22*). Cohesin loops at individual loci appear to be transient in nature and relatively rare events, residing in a loop conformation <10% of the time in a single locus imaged in mouse embryonic stem cells (*23*). Last, TAD boundaries appear to be crucial elements in collective genome function as their targeted knockout results in pathogenic phenotypes during mouse development (*24*). As TADs arise in population measurements, it was proposed that TADs are an ensemble feature of cellular population measurements; as such, they may represent infrequent but crucial structures necessary for proper collective function (*17*, *23*). Prior modeling has suggested that TADs arise from variations in loop domain events within a population, suggesting that TADs and loops are potentially convergent structures in single cells.

In support of this, higher-order structures have been observed with variable positions and frequencies using oligopaint–based super-resolution microscopy (*17*, *20*–*22*), and loss of RAD21 did not disrupt chromatin cores and nanodomains on structured illumination (*22*, *25*). Despite the association of TADs with cohesin-mediated loop extrusion, RAD21 depletion did not result in the loss of all the TAD-like structures, nor did it alter the structure of nanodomains in individual cells (*14*, *17*, *20*–*22*). Furthermore, the boundary strengths of TAD-like structures were comparable in the RAD21-depleted cells to those with intact cohesin (*20*, *22*). Although oligopaint–based super-resolution imaging provides sequence-specific loci information, the resolution limit of this modality approaches ~30 nm and can only target specific loci for study (*14*, *17*, *20*–*22*). Furthermore, these methods rely on the formamide-based DNA denaturation, which alters the nanoscale 3D genome structure on electron microscopy (*26*), with recent work demonstrating that formamide denaturation approaches cause irreversible swelling of the chromatin chain and collapse of supranucleosomal structure. As such, oligopaint alone cannot provide insights on the transition of chromatin from the disordered polymer structures observed via chromatin electron tomography (ChromEMT) into higher-order structures. Likewise, it is insufficient for providing quantitative information about the global features of chromatin 3D folding throughout the nucleus (*27*–*29*).

In their seminal work, Ou *et al.* (*27*) demonstrated that by using photooxidation of a DNA-specific dye, osmium tetroxide could be specifically localized to DNA in a density-dependent manner. With this approach, ChromEMT was able to overcome the limitation from prior EM studies of chromatin and allow the direct resolution of DNA, nucleosomes, and fibers. In chromatin electron microscopy (ChromEM) imaging, mass density is proportional to intensity. As a result, unlike super-resolution imaging techniques with comparable resolution, ChromEM resolves the ground-truth structure of chromatin (*27*). Combining ChromEM with scanning transmission chromatin electron microscopy (ChromSTEM) with high-angle annular dark-field (HAADF) tomography, we previously demonstrated that we can thus resolve chromatin structure with a resolution of 2 nm and allow direct quantitative analysis of chromatin density across all the regulatory length scales of the genome in 3D (*28*, *29*). Using ChromSTEM-HAADF tomography, we demonstrated previously that chromatin organization transitions from the disordered polymer structure observed in the study of Ou *et al.* into higher-order packing domains (PDs) that are 50 to 200 nm in size and composed of ~100 to 500 kb of DNA; sizes which were remarkably similar to those predicted for TADs and loops (*28*, *29*). Whether packing domains form primarily due to loop extrusion processes and how TADs relate to ChromEM-resolved higher-order chromatin structures evades our understanding.

To address these questions, we performed ChromSTEM tomography on RAD21-depleted cells using the HCT116 RAD21-mAID-Clover CMV-osTIR1(F74G) cell line. We find that although packing domains contain comparable DNA content to TADs and loops, they are not the physical manifestation of these structures. Instead, our findings indicate that cohesin helps with the formation of nascent domains (small, lower-density packing domains) by forming long-range interactions (TADs and loops) as half of are predominantly lost in ChromSTEM tomography. By pairing these results with multicolor single-molecule localization microscopy (SMLM) with chromatin interaction analysis by paired-end tag (Chia-PET) sequencing, transposase-accessible chromatin with high-throughput sequencing (ATAC-seq), and chromatin immunoprecipitation sequencing (ChIP-seq), we show collectively that the act of creating long-range connections likely produces nascent domains. We then show that domain maturation occurs from nucleosome posttranslational modifications such as histone deacetylation mediated by histone deacetylase 3 (HDAC3). In summary, by pairing ChromSTEM tomography and SMLM with multiomic analysis, this work demonstrates that (i) packing domains are not the physical correlate of TADs and (ii) how cohesin-mediated and nucleosome-mediated processes intersect for domain formation and maturation to occur.

## RESULTS

### Chromatin organizes into three distinct regimes in colonic HCT-116 cells

ChromSTEM has previously demonstrated that chromatin organization exists across three hierarchies in A549 pulmonary epithelial cells and in BJ fibroblast cells: (i) a disordered chromatin polymer (5 to 25 nm), a power-law polymer (50 to 150 nm), and a space-filling territorial polymer (>200 nm) (*28*, *29*). With respect to the disordered polymer, these observations were consistent with the structures previously identified by Ou *et al.* (*27*) where chromatin organizes as a flexible fiber with variations in density and folding. This disordered polymer produces a power-law polymeric regime at higher length scales due to the resulting formation of packing domains. Last, these packing domains converge into a territorial polymer with a random spatial arrangement of densities. Notably, this last regime is not an assembly of the underlying regimes but is a random distribution of mass density (*28*, *29*).

**Fig. 1.**
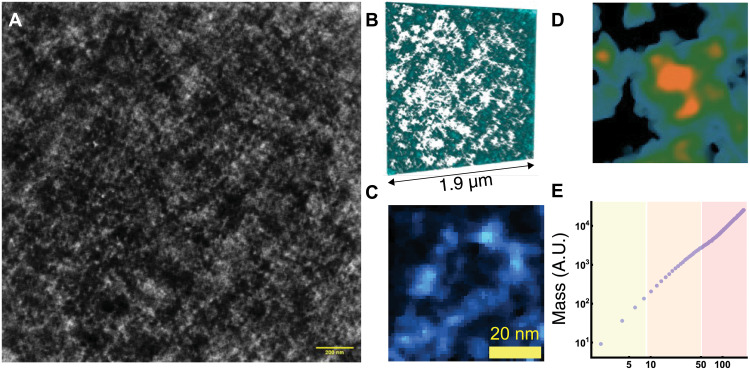
ChromSTEM preparation and tomogram analysis. (**A**) High-resolution mean projection from ChromSTEM in HCT-116 cells. (**B**) Tomogram reconstruction showing distribution of high-density areas with surrounding porosity in a 100-nm section. (**C**) Visualized chromatin loop with an approximate length of 120 nm. (**D**) High resolution of packing domain tomogram projection (200 nm by 200 nm) showing high density within chromatin center with progressively decreasing intensity until porous regions are encountered (black). (**E**) Log-log plot of mass density distribution versus radius from the visualized packing domain demonstrating the emergence of three distinct chromatin regimens: yellow (disorder polymer), orange (power-law polymer), and red (territorial polymer). The transition between states occurs throughout the nucleus and varies between packing domains. A.U., arbitrary units.

To investigate whether this organization further extends in human cells to HCT-116 colonocytes, a model of microsatellite unstable colorectal cancer, we performed ChromSTEM-HAADF tomography using the protocol previously described (*28*, *29*). Briefly, a major prior limitation of electron microscopy within the nucleus was the result of nonspecificity of negative staining agents to both chromatin and nonchromatin molecules within the nucleus. To overcome this limitation, Ou *et al.* (*27*) demonstrated that utilization of the DNA-specific dye, Deep Red Anthraquinone 5 (DRAQ5), with photooxidation results in the preferential binding of osmium of DNA. DRAQ5-stained HCT-116 control cells were resin embedded, and cell nuclei were identified using wide-field optical microscopy. We subsequently sectioned 120-nm resin section and performed dual-tilt STEM with HAADF imaging on a 1.9 μm–by–1.9 μm section producing a high-resolution tomogram from within the center of the nucleus (Fig. 1, A and B). As expected, ChromSTEM tomography resulted in the reconstruction of high-resolution features of chromatin including 3D-rendered individual fiber loops (Fig. 1C and movie S1) and chromatin packing domains (Fig. 1D and movie S2). Consistent with prior studies in A549 and BJ fibroblast cells, chromatin organized into three regimes within HCT-116 control cells with a power-law like geometry within supranucleosomal length scales apparent (Fig. 1E) within individual domains.

### Packing domains are heterogeneous, higher-order supranucleosomal structures

**Fig. 2.**
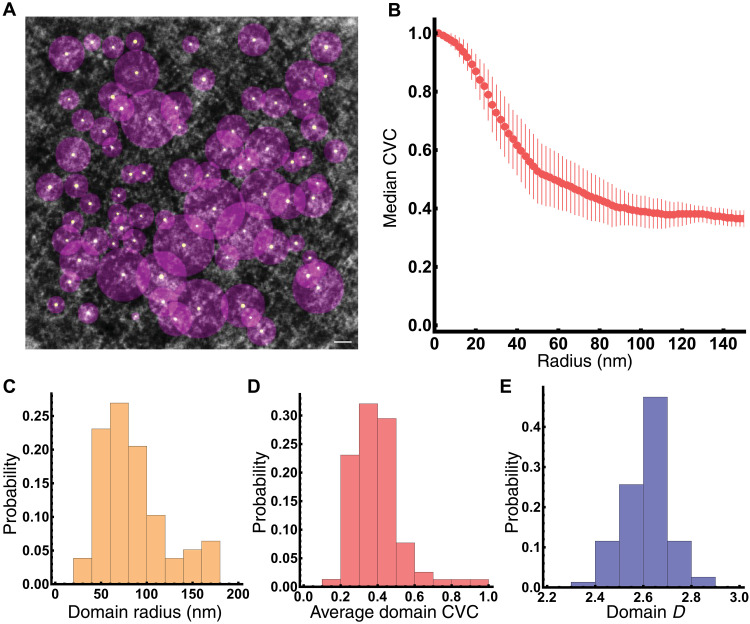
Chromatin organizes into packing domains in HCT-116. (**A**) Packing domains identified in ChromSTEM tomogram with projection of their centroid (white circle) and bounding radius (purple) in control cells. Scale bar, 100 nm. In total, 78 packing domains were identified within this tomogram. Packing domains are identified by local threshold detection, and the radius is determined by the minima of either the (i) radius deviates from log-log density, (ii) the radius that reaches the first derivative, or (iii) the radius at which the minimum density occurs. (**B**) Chromatin volume fraction decays from the center of packing domains toward their periphery, with the PD density approaching the average CVC of the nucleus near the periphery of the packing domains. Error bars represent median deviation. (**C** to **E**) Packing domains are heterogeneous structures with a distribution of sizes (C), CVC (D), and power-law packing (E).

Using the produced ChromSTEM tomogram, we subsequently performed analysis to evaluate the organization of structure into packing domains. In prior work, it was shown that packing domains are heterogeneous structures with a distribution of sizes, densities, and packing efficiency. A characteristic feature of packing domain organization is that the density distribution follows a power-law geometry characteristic to polymeric structures such as chromatin that is detailed below. To identify packing domains, we first analyze the mean projection of the ChromSTEM tomogram to identify the loci with the highest local density as defined by the 1.5 times the SD of the local density. The size of packing domains is then defined by the smallest radius from three independent classification measurements: the radius where deviation of density from the log-log density-size distribution occurs (i.e., where it no longer follows power-law mass density behavior), the radius at which the first derivative reaches a space-filling geometry, and the radius at which the minimum of density occurs (Fig. 2A and fig. S1). In this approach, packing domains (pink circles) with their local centers (white points) are identified for further analysis (Fig. 2A).

We observe that packing domains demonstrate the highest density within the center [chromatin volume concentration (CVC), ~1 at the interior core] with the subsequent decay to the nuclear wide average CVC of ~0.3 (Fig. 2B). As expected, PDs have a distribution of sizes (Fig. 2C, average radius of 84 nm with an SD of 36 nm) and average chromatin volume fractions (Fig. 2D, average CVC of 0.40 with an SD of 0.13). As a polymeric structure, the relationship between the mass density distribution of chromatin and its shape can be quantified either by the contact scaling relationship, *S*, or in relation to the mass-distance relationship with fractal dimension, *D*, as follows. *S* measures the frequency of contacts between “monomers” in relation to the linear distance on the chain and decays as a function of distance depending on solvent conditions, confinement, crowding, and other considerations. *D* is a complementary measure of the polymer which relates the distribution of mass to the occupied volume as a function of the radial distance, *r*, by *M* ∝ *r^D^*. As with *S*, the measured *D* for a chromatin polymer depends on the solvent conditions, monomer-monomer interactions, crowding, etc. It is well established that chromatin is a complex polymer, and its function is thought to arise from how it is folded within space. However, while it is known that it is not in the limiting case of a collapsed, space filling globule structure where *D* = 3 nor is it a polymer in idealized solvent conditions where the monomers prefer interactions with the solvent where *D* = 5/3; the exact configurations of chromatin in 3D space across conditions and length scales is an area of active research. It has been observed in multiple experimental modalities that chromatin is a power-law polymer where interactions with the monomers are preferred resulting in *D* ranging between 2 and 3 at supranucleosomal length scales.

Using the relationship between mass and distance, we calculated *D* within packing domains and found that, as expected, they deviate from a purely space-filling geometry with an average *D =* 2.61 (SD of 0.09; Fig. 2E) in control HCT116-RAD21-auxin-inducible degron 2 (AID2). In conjunction with prior studies, this suggests that supranucleosomal chromatin organization is not simply a polymer in a good solvent (*D* = 5/3), nor does it resemble a purely space-filling globule (*D* = 3) but is instead a disordered polymer assembly with a maximum observed *D* of 2.84 (Fig. 2E). A notable feature of packing domain structure is the corrugation, indicating the continuous distribution of chromatin transitions from chromatin dense regions to porous open segments (Fig. 1D) that could be accessible to larger enzymatic machinery for molecular functions such as gene transcription. Overall, these findings were consistent with the findings in A549 lung epithelial cells and BJ fibroblasts, indicating that packing domains may further generalize as the intermediate functional hierarchy of chromatin within human nuclei.

### Chromatin packing domains are largely stable despite RAD21 depletion

**Fig. 3.**
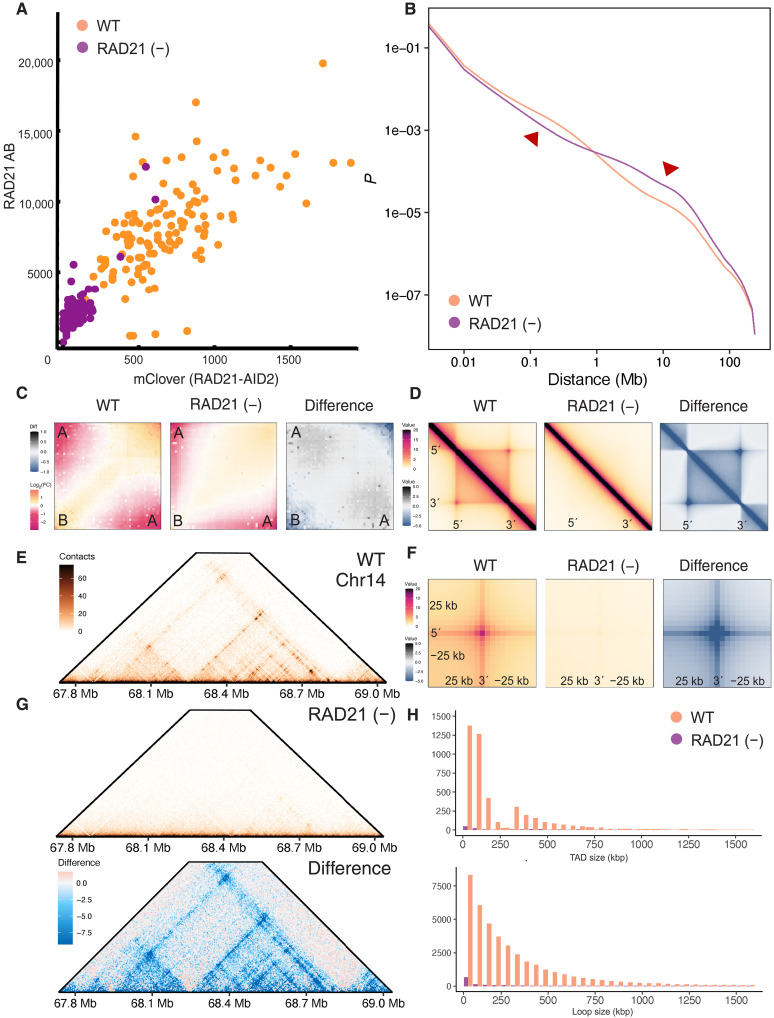
RAD21 depletion results in expected loss of TADs, loops, and decreased insulation. (**A**) Verification of RAD21 depletion by both quantification of mClover and RAD21-immunofluorescent staining showing that more than 90% of the population is without detectable RAD21. Axes representing corrected total fluorescence in arbitrary units. (**B**) Contact scaling is observed to decrease at short ranges and increase at long ranges upon RAD21 depletion. (**C**) Compartment insulation demonstrates compartment score weakening with RAD21 depletion. (**D**) TAD insulation plots demonstrating TAD insulation strength decreases with RAD21-depletion. (**E**) Representative loop domain anchor point on chromosome 14 showing that while the loop domain in control cells. (**F**) Observed weakening of loop anchors on the insulation plot. (**G**) Comparison of loop domain observed in (E) upon RAD21 depletion demonstrating a decrease in local contacts. (**H**) Quantification of TADs and loop domains observed in control versus RAD21(−) cells showing predominantly a loss of >95% of TADs and ~95% of loop domains at 6 hours of treatment with 1 μM 5-Ph-IAA. WT, wild type; FC, fold change.

Given the comparable DNA content between packing domains and TADs, we wanted to understand whether (i) packing domains correlated with TADs or loop domains and (ii) whether impairment of cohesin-mediated loop extrusion weakened the boundaries or sizes of packing domains proportional to the decrease observed on ensemble measures, such as Micro-C. To investigate the role of cohesin-mediated loop formation on packing domain structure, we used the AID2 system in HCT-116 cells to rapidly degrade endogenous RAD21 and analyzed publicly available high-resolution Micro-C libraries available through the Encyclopedia of DNA Elements (ENCODE) Consortium as previously described (*12*, *30*–*34*). We chose this cell line due to the superiority in preventing leakage-associated loss of the RAD21, the increased rapidity of degradation, and the decreased cytotoxicity (*30*). Given these features and the higher resolution of Micro-C, analysis of the RAD21’s effect on genome connectivity could provide insights on the role of the crucial protein, RAD21, in cell function and serve as a positive control for ChromSTEM imaging. To verify depletion of RAD21 following 6 hours of 5-phenyl-indole-3-acetic acid (5-Ph-IAA) treatment, RAD21 protein levels were quantified independent of the degron construct using confocal microscopy (Fig. 3A and fig. S2) demonstrating complete depletion of RAD21 in >90% of the cell population by both immunofluorescence and mClover levels (Fig. 3A). Using publicly available data through the ENCODE Consortium (*32*–*34*), we then analyzed the effect of RAD21 depletion on TADs, loop domains, compartments, and genome connectivity in Micro-C (accession numbers are available in table S1). As expected, we verified that RAD21 depletion results in the marked decrease of TADs and loops, with a visually apparent transformation in contacts genome wide (Fig. 3, B to H) (*13*, *15*, *35*, *36*). Aligning with previous results, we observed a robust decrease in compartment strength (Fig. 3C and fig. S3) alongside increased compartmentalization and some compartment switching from A to B, which was previously seen in NIPBL mutant mice (*15*, *37*). Consistent with prior reports demonstrating complete loss of TADs and loops, we observed that RAD21 depletion within RAD21-mAID-Clover CMV-osTIR1(F74G) cells was robust. In particular, we observed that ~97% of all TADs were lost (Fig. 3, D and H, and fig. S3). Likewise, we observed that ~95% of all loops observed in the control condition were no longer present (Fig. 3, E to H, and fig. S3).

**Fig. 4.**
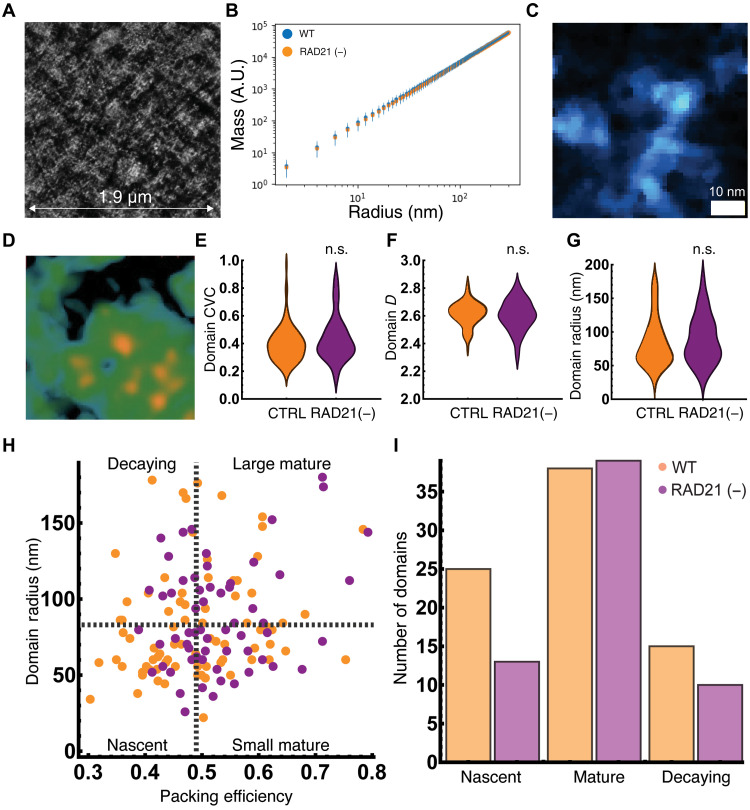
Packing domain size and organization are minimally transformed upon RAD21 depletion. (**A**) ChromSTEM tomogram from RAD21 5-Ph-IAA–treated cells after 6 hours of depletion demonstrating remarkable similarity to chromatin DMSO control cells above. (**B**) Analysis of chromatin density distribution demonstrates minimal changes in chromatin organization across all three regimes. (**C**) Visualized chromatin loop in RAD21-depleted tomogram demonstrating the continued presence of long-range interactions despite loss of cohesin-mediated loop extrusion. (**D**) Representative packing domain (200 nm by 200 nm) from RAD21-depleted cells showing similar features to those within control cells with a high-density center and continuous distribution of mass toward the periphery until the emergence of low-density porous regions. (**E** to **G**) Overall, there was a total decrease in the number of observed packing domains from 78 to 62 upon RAD21 depletion; however, the remaining domains had similar CVC (E), scaling of packing (F), and size (G) in both conditions. *P* values represent two-tailed unpaired *t* test from the 78 and 62 packing domains. Bonferroni corrections were assumed for multiple comparisons (total of three) for an adjusted *P* value of 0.0166. (**H**) Analysis of packing domains by size and packing efficiency to analyze domain properties. Nascent domains (low efficiency and small size), mature domains (high packing efficiency), and decaying domains (low efficiency and large size) are differentially affected upon 1 μM ph-IAA for 6 hours. Black dashes represent mean packing efficiency and mean radius in control cells. (**I**) The primary decrease in packing domains occurred in nascent domains (low-density, small domains; *n* = 25 to 13, ~48% decrease) with a negligible change in the number of mature domains (high efficiency, *n* = 38 to 39, ~2% increase). Decaying domains were also decreased (low efficiency and large size; *n* = 15 to 10, 33% decrease). n.s., not significant.

Having verified that (i) RAD21 was depleted throughout the population at short-time scales and (ii) ensemble Micro-C demonstrated that TADs and loops were lost upon RAD21 depletion, we compared these findings to the structure of chromatin in ChromSTEM tomography of RAD21 cells treated with 1 μM 5-Ph-IAA for 4 hours. In contrast to the findings on Micro-C, the ChromSTEM tomogram generated upon RAD21 depletion was visually very similar to those in dimethyl sulfoxide (DMSO)–treated control cells, demonstrating a continuous distribution of heterogeneous structures and porosity throughout the examined field of view (Fig. 4A). Furthermore, the mass-scaling relationship observed in DMSO controls with the observation of three distinct chromatin regimes was not lost upon RAD21 depletion (Fig. 4B) and was comparable to that observed in the DMSO controls. With respect to both the disordered chromatin polymer (<25 nm) and packing domains, RAD21 loss did not have a proportional effect on 3D genome organization to that observed on Micro-C. At the level of chromatin as a disordered polymer, we observed the existence of looping chromatin fibers (Fig. 4C and movie S3), indicating that long-range interactions persisted.

Performing the analysis to identify packing domains as described above upon RAD21 depletion, we did observe an overall decrease in the number of packing domains (from 78 in DMSO-treated cells to 62 in RAD21-depleted cells, ~20% decrease), but the remaining domains were visually comparable to those observed in controls (Fig. 4D and movie S4). Quantitatively, RAD21 loss only had a modest effect on the remaining packing domains, with small changes in CVC (0.40 to 0.43, *P* value of 0.20; Fig. 4E), *D* (2.61 to 2.60, *P* value of 0.45; Fig. 4F), and radius (84 nm to 89 nm, *P* value of 0.39; Fig. 4G). To test whether these findings extended into live cells and were not due to transformation from fixation, we performed live-cell partial-wave spectroscopic (PWS) microscopy which can measure changes in *D* for chromatin between 20 and 200 nm for control cells in comparison with that of RAD21-depleted cells (*29*, *38*). Comparable to the findings on ChromSTEM microscopy, we observed a minimal change in *D* in live cells upon 4 hours of RAD21 depletion (2.61 à 2.60, *P* value of 0.104; fig. S4), indicating that chromatin organization is predominantly preserved despite loss of RAD21 both on electron microscopy and in live cells.

### RAD21 depletion affects half of nascent chromatin packing domains

A critical feature of ChromSTEM tomography is the ability to resolve and quantitatively characterize all structures above individual nucleic acids given the nominal resolution of 2 nm (*28*, *29*), allowing study of genome organization that cannot be probed even by super-resolution optical techniques. Although ChromSTEM imaging is restricted to fixed cells, in principle, the observed chromatin structures capture a temporal cross section of evolving structures. As such, one could consider that the size and density distribution of packing domains could relate to their current progress through a packing domain life-cycle. In such an analysis, dense structures represent mature domains, whereas small, low-density structures represent nascent domains, and lastly, large, low-density structures could represent decaying domains. Since our results indicated that packing domains were not the manifestation of TADs, an alternative hypothesis is that cohesin-mediated looping could either be involved in barrier maintenance to prevent domain overlap or involved in domain formation (*20*–*22*). To address this using ChromSTEM, we investigated the effect of RAD21 depletion on these subpopulations.

We partitioned the features of packing domain across their size in comparison to their packing efficiency (*28*), which measures the per-unit area density of chromatin in the ChromSTEM tomogram (Fig. 4H). Packing efficiency ranges from 0 to 1, with a value of 1 corresponding to an optimal space filling configuration where DNA fully fills the space across the domain volume. In this analysis, nascent structures are small and poorly packaged (below average size and below average packing efficiency in control tomogram), mature structures can be large or small but are efficiently packed (above average efficiency), and decaying structures are large, poorly packed domains (above average size and below average efficiency). Notably, loss of RAD21 results in the largest change in the proportion of nascent domains (*n* = 13 versus *n* = 25 in controls, ~48% decrease in number), whereas there was a minimal effect in the number of mature domains (*n* = 39 versus *n* = 38 in controls, ~2% increase; Fig. 4, H and I). In the context that RAD21 is depleted within 1 hour in this cell line model (fig. S5) (*30*), this indicates that mature domains do not depend on cohesin function for continued stability. Furthermore, that half of nascent domains were still observed at 4 hours indicates the presence of a secondary mechanism that induces domain formation. It is also worth noting that ChromSTEM imaging captures the statistics of single-cell organizational states, whereas the ensemble measurement of Hi-C is an aggregate statistic of many organizational states. Consequently, this suggested that mature packing domains were largely maintained independent of cohesin-mediated loop formation but that the principal effect of RAD21 extrusion was on the production of a subset of nascent domains. Although the stability of mature domains is possibly consistent with findings in prior reports using oligo-based methods (*20*, *22*), visualization and quantification of the distribution of domain structures cannot be achieved with these prior methods due to resolution limits.

**Fig. 5.**
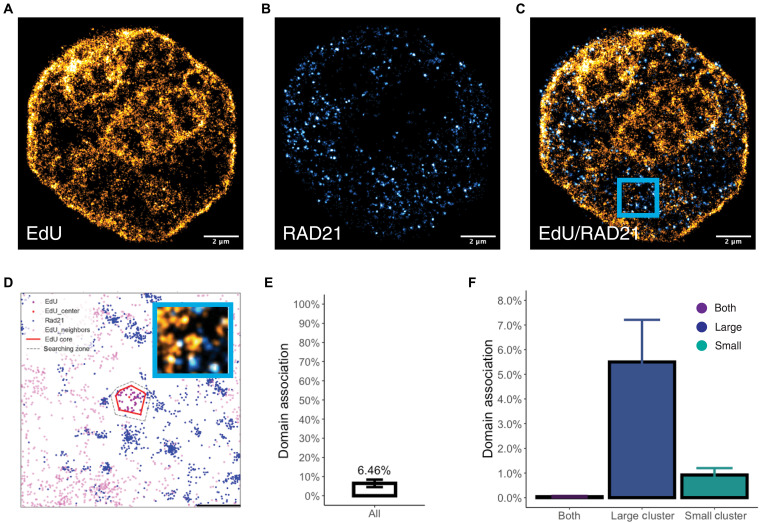
RAD21 primarily localizes in regions of low DNA density. (**A** to **C**) SMLM microscopy of RAD21 localization with DNA as stained by EDU. (A) Representative nuclear image of DNA stained with EdU with a localization average uncertainty of ~30 nm. (B) Representative image of RAD21 with SMLM with an average uncertainty of ~30 nm. (C) Overlay from two channels showing that RAD21 primarily localizes in regions devoid of large DNA domains. (**D**) Representative spatial analysis from two-color SMLM in (A) to (C) with a bounding region of 60 nm to measure the frequency of RAD21 (blue) association with DNA (purple) domains. Scale bar, 500 nm (**E**) Quantification of the frequency of RAD21 localizations associating with DNA domains in imaged nuclei (average, ~6.46%; *n* = 12). (**F**) Subgroup analysis of the type of domain RAD21 is within a 60-nm boundary of a domain showing that it primarily associates with larger domains (>80 nm) compared to small domains (20 to 80 nm). Analysis is from *n* = 12 cells, *P* value < 0.05. Rarely, RAD21 events were found at the intersection of large and small domains (both, <1%).

Given these findings, we hypothesized that if mechanistically RAD21 intersects with domain formation, it would primarily localize in low DNA density regions to help facilitate assembly. Since multimolecular labeling cannot be fully addressed by ChromSTEM tomography at this time, we instead used two-color SMLM that has a positional uncertainty of ~30 nm using antibody-conjugated staining to detect RAD21 positioning and 5-ethynyl-2′-deoxyuridine (EdU) staining to stain for DNA. Accounting for a major limitation in SMLM that density is not fully proportional to the number of emission events (each dye molecule has variations in emission frequency cycles, and penetration can be nonuniform depending on dye size within high-density structures such as domains), we focused on comparing how likely RAD21 was to localize near DNA-rich domain-like structures. Therefore, although SMLM in this analysis cannot distinguish between high and low packing efficiency domains, the distribution of RAD21 relative to domain-like spaces or domain-less spaces can be functionally informative in the context of the findings on ChromSTEM. Consistent with the hypothesis that RAD21 helps with domain assembly, we observed that RAD21 primarily localizes into areas with low DNA density (Fig. 5, A to C). Quantitatively, only 6.5% of RAD21 colocalizes with domain-like structures within 60 nm of the domain boundary (Fig. 5D, interquartile range from 0.7 to 10.5%, *n* = 12 cells). Analyzing the distribution of RAD21 as a function of domain size, RAD21 predominantly localized near larger domains (Fig. 5E) with an average of 5.5% (>80 nm, interquartile range of 0.4 to 8.3%) compared to mean 0.9% for small domains (20 to 80 nm, interquartile range of 0.01 to 1.0%, *P* value of 0.014). This low frequency of association was comparable to findings of the brief lifetime of loops and TAD engagement by cohesin in live-cell imaging reported. When paired with the stability of the remaining domains observed on ChromSTEM imaging and the loss of half of nascent domains, the scarcity of RAD21 with domains on SMLM suggests that a major function of RAD21 is in domain creation.

### Packing domains are not the physical manifestation of TADs

**Fig. 6.**
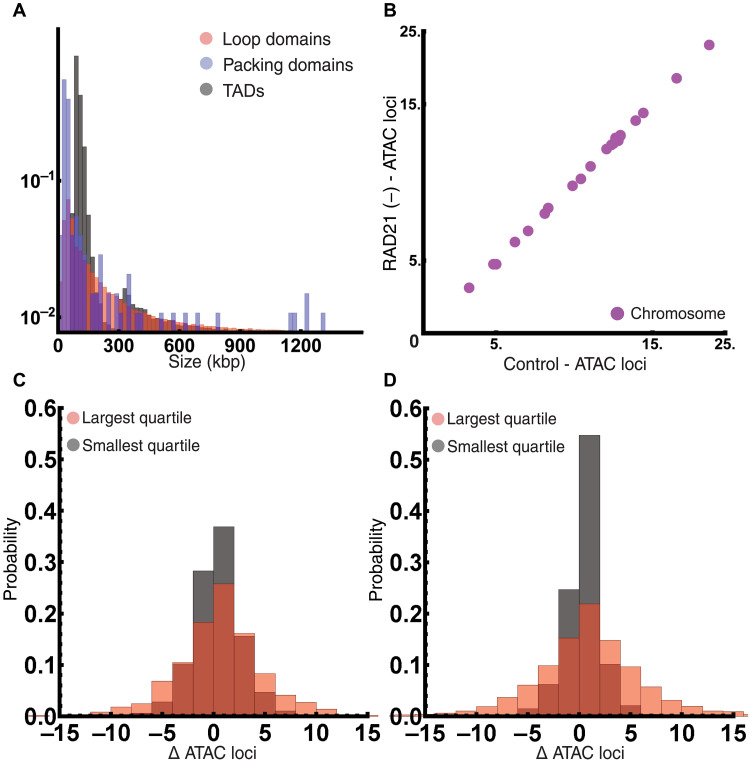
Analysis of TAD and loop domains indicates no significant change in global or local ATAC-seq loci with cohesin depletion. (**A**) Comparison of packing domain DNA content (median, 89.32 kbp; minimum of 6.27 kbp and maximum of 1.306 Mbp) in comparison to loop domains (median, 170 kbp; minimum of 9 kbp and maximum of 9.06 Mbp) and TADs (median, 130 kbp; minimum of 60 kbp and maximum of 2.55 Mbp) showing similar ranges in size in control cells. (**B**) Analysis of ATAC-seq accessibility per chromosome in control cells versus RAD21(−) cells showing no significant change in global accessibility. Axes are reported in number of loci times 10^3^. (**C**) Analysis of the change in number of ATAC-seq loci within TAD coordinates before and after RAD21 depletion showing no change in accessibility in TAD regions upon their loss (median = 0, interquartile range of −2 to +2). (**D**) Analysis of the change in number of ATAC-seq loci within loop domain coordinates before and after RAD21 depletion showing no change in accessibility in loop regions upon their loss (median = 0, interquartile range of −2 to +2).

The retention of the majority (~80%) of supranucleosome packing domains contrasted sharply to the loss of ~97% of TADs (Figs. 3, D to H, and 4, H and I) and 95% of loop domains (Figs. 3, D to H, and 4, H and I) upon RAD21 depletion. Considering recent work demonstrating that there is a distribution of transient loops and TAD anchors observed in live cells, this disparity between connectivity and structure could be explained by the fact that not all nascent structures are converted into mature domains in the exact manner. This observation raised an interesting question: If long-range interactions allocate genomic content into nascent structures, then what mechanisms are necessary to facilitate domain maturation? This leads us to hypothesize that cohesin functions in the allocation of DNA by defining distal interactions, which may then be modified by additional mechanisms to produce distinct physical configuration. In this hypothesis, although TADs and loops are distinct in the population ensemble, cohesin function in individual cells is to define genomic allocations. First, we verified that packing domains were comparable to TADs and loop domains at the level of their genomic content to ensure that packing domains were not sub-TAD or subloop structures (Fig. 6A). The genomic content within packing domain sizes ranged between 6 kbp and 1.3 Mbp in control cells (median size of 89 kbp) in comparison to TADs with a range of 60 kbp to 2.5 Mbp (median size 130 kbp) and loop domains ranging from 9 kbp to 9 Mbp (median size of 170 kbp), indicating that these were comparable structures. Next, we tested whether TADs and loop domains have similar changes in accessibility and nucleosome posttranslational modification upon RAD21 depletion. Specifically, if TADs and loops are manifestations of packing domains, then they would have similar changes: (i) an increase in general accessibility within these loci and (ii) a decrease in nucleosome modifications associated with high density [e.g., a decrease in histone 3 lysine 9 trimethylation (H3K9me3)].

**Fig. 7.**
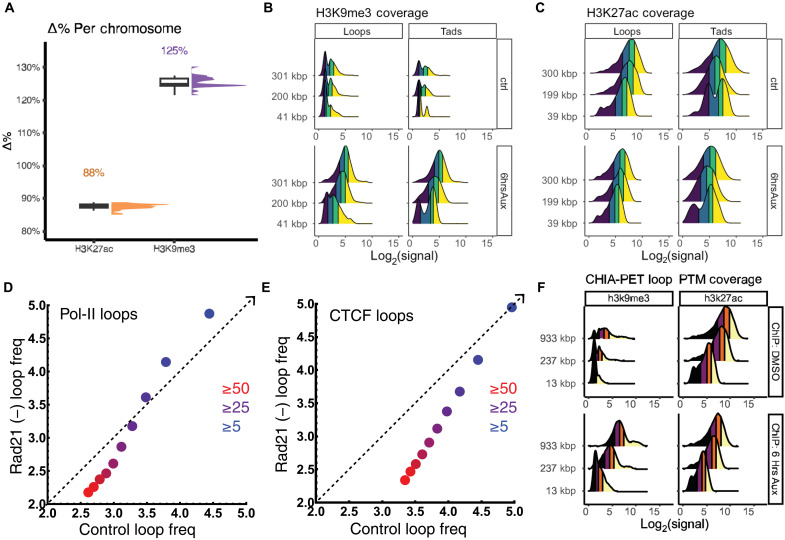
Analysis of nucleosome modifications with RAD21 depletion. (**A**) RAD21 depletion is associated with a chromosome-wide accumulation of H3K9me3 (23% increase) and a decrease in H3K27ac (13% decrease) in a population ensemble. (**B** and **C**) Comparison of loop domains and TADs to RAD21 depletion demonstrating a parallel transformation. (B) H3K9me3 marks increase, predominantly within longer loops and TADs. (C) H3K27ac marks decrease independent of loop or TAD length. (**D**) Chia-PET Pol-II–mediated loops as a function of interaction frequency demonstrating stability to RAD21 depletion. *X* and *Y* axis scales represent log_10_ number of loop events. (**E**) Chia-PET of CTCF-mediated loops as a function of interaction frequency demonstrating loss upon RAD21 depletion at the level of nucleosome posttranslational modifications. *X* and *Y* axis scales represent log_10_ number of loop events. (**F**) Analysis of H3K9me3 and H3K27ac loci within Pol-II loops demonstrating that, phenotypically, they behave similar to RAD21-mediated loops.

To test this hypothesis, we used publicly available ATAC-seq data from the ENCODE Consortium (*32*–*34*) on RAD21-depleted AID2 cells (accession numbers are in table S2). Although ATAC-seq is limited in that it cannot fully probe the finer-scale features of the chromatin chain, it instead is used to provide general measurements of accessibility. On analysis of ATAC-seq, we found no change in the frequency of accessible loci per chromosome with or without RAD21 depletion at 6 hours (Fig. 6B). This indicated that the global change in connectivity does not result in global alterations in general chromatin accessibility. Next, we tested whether local accessibility differentially changed within TAD loci or loops. If these were distinct space-filling domains, then general changes in local accessibility upon the loss of these structures would occur. We again found no change in the general accessibility in either TADs (Fig. 6C and fig. S6) or loop domains (Fig. 6D and fig. S7) upon RAD21 depletion independent of their size. We then examined whether they have similar associations and transformations in nucleosome posttranslational modifications before and after RAD21 depletion. It was previously demonstrated by Rao *et al.* (*13*) that loops have distinct transformation in histone 3 lysine 27 acetylation (H3K27ac) and H3K9me3 depending on the temporal behavior of loop domains. As our data represent one temporal cross section, we focused on comparative analysis by loop and TAD size. We find that independent of their size, TADs and loop domains have similar transformations in these nucleosome posttranslational modifications after RAD21 depletion (Fig. 7, A and B). We observed that H3K27ac decreases, whereas there is a length-dependent increase in the presence of H3K9me3 within both TADs and loops (Fig. 7, A and B). The expansion of H3K9me3 within TADs and loops, coupled with our observations that RAD21 is concentrated in DNA-poor regions (Fig. 5) and that RAD21 degradation leads to loss of nascent domains (Fig. 4), supports the hypothesis that cohesin primarily functions through allocation of DNA.

Considering that TADs and loops share the similar behaviors and that even with the loss of RAD21, ~50% of nascent domains are observed, we hypothesized that another loop forming processes such as transcription could account for a second method of nascent domain formation. If this were the case, then genome allocations produced by RNA polymerase II (Pol-II)–mediated loops would resemble the loops formed by RAD21. However, these loops would continue despite the elimination of RAD21. To test this hypothesis, we used Chia-PET data available on ENCODE to measure the behavior of RNA Pol-II loops before and after RAD21 depletion. As expected, we found that RAD21 depletion had a minimal impact on Pol-II loops. Likewise, the nucleosome composition of these loops was similar at the level of H3K9me3 and H3K27ac (Fig. 7, C and D). Paired with prior work showing that heterochromatin dense structures are stable after RAD21 loss (*25*), this suggests that although TADs and loop domains are distinct connectivity features in a population ensemble, these may not translate into separate processes in domains observed on ChromSTEM.

### HDAC3 inhibition results in accumulation of nascent domains

We next set out to understand what mechanisms result in domain maturation. By definition, DNA within mature domains is more efficiently packed than in nascent or decaying domains. As a result, chromatin within mature domains requires more tightly packed nucleosomes. Our results showed that DNA segments formed into loops and TADs had accumulation of H3K9me3 after RAD21 loss. This suggested that RAD21 plays a role in domain formation by defining recurrent DNA within the cell population to form into nascent domains. Furthermore, even as RAD21 establishes genomic segments into nascent domains, this alone does not dictate the end type of maturation (i.e., into one large domain, several small domains, or disintegration). In the absence of RAD21, other mechanisms can still produce nascent domains but potentially with less precision across the population. Nascent structures produced by other processes (potentially transcription for example) will maintain their respective stability and continue to produce such allocations. However, because of this decreased precision, variable DNA segments are then potentially exposed to heterochromatin enzymes. In a population measurement such as ChIP-seq, these variations in position result in ensemble peaks to be present. However, in individual cells, the frequency of heterochromatin formation and the number of heterochromatin structures remain stable.

**Fig. 8.**
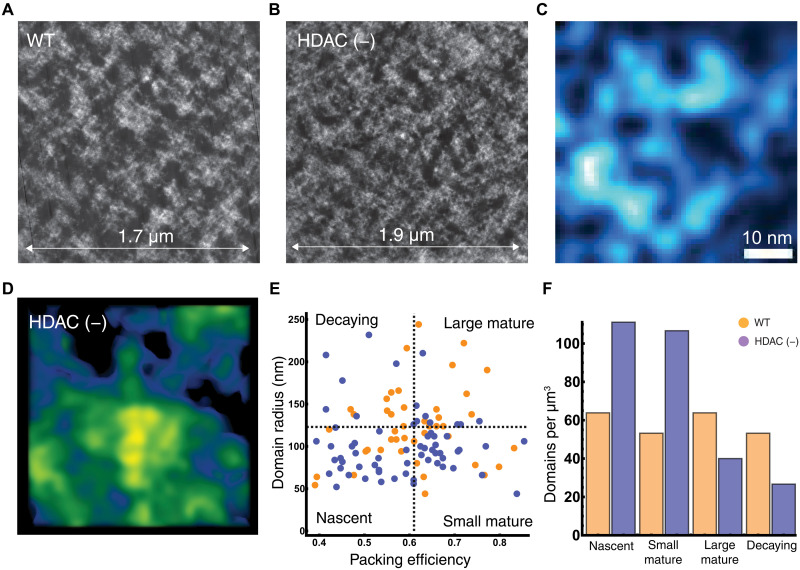
HDAC3 inhibition is associated with impaired packing domain maturation and accumulation of nascent domains. (**A**) Representative tomogram from control HCT-116 cells compared to (**B**) HCT-116 cells treated with 1 μm of Rgfp966 for 24 hours to inhibit HDAC3 function. Visually, packing domain structures have decreased in size. (**C**) Representative visualized loop of DNA in Rgfp966-treated cells. (**D**) Representative packing domain from Rgfp966-treated cells demonstrating expansion and decreased core density. (**E**) Quadrant analysis of the respective domain types in control versus HDAC3-inhibited cells. (**F**) Analysis of chromatin packing domains demonstrating an accumulation of nascent domains (74% increase) and small mature domains (100% increase). Frequency of domains was adjusted to per cubic micron due to differences in the visualized sample area in control compared to HDAC3 inhibition. Concurrently, there is a decrease in the number of large, mature domains (37% decrease). This overall suggests that heterochromatic nucleosome posttranslational modifications help facilitate domain maturation and consolidation.

Using ChromSTEM tomography, we therefore tested the hypothesis that heterochromatin-modifying enzymes, such as HDAC3, are necessary for domain maturation to occur. If HDAC3 is necessary for domain maturation, then we hypothesized that there would be an accumulation of nascent domains with HDAC3 inhibition. Since other nucleosome posttranslation processes remain intact, we would not observe a complete disintegration of mature domains. Visually, we observe that with HDAC3 inhibition, there is a loss of large, high-density domains throughout the tomogram compared with the control (Fig. 8, A and B). Consistent with the loop formation being independent of heterochromatin remodeling, loop structures were visualized in the setting of HDAC3 inhibition (Fig. 8C). Individual packing domains were less likely to have high-density cores, but instead mass was diffusely expanded (Fig. 8C). Consistent with the hypothesis that nucleosome posttranslational modifications facilitate domain maturation, HDAC3i was associated with an accumulation of nascent domains (74% increase; Fig. 8, E and F) and small mature domains (~100% increase; Fig. 8, E and F). Conversely, large mature domains were less likely to form upon HDAC3 inhibition, and we observed a ~37% decrease in these compared to controls (Fig. 8, E and F). This observation of differential effects on small mature and nascent domains compared to large mature domains suggests that once a nascent domain is created, the end transformation depends on other processes such as nucleosome modification. Furthermore, the accumulation of small but mature domains indicates that heterochromatin enzymes facilitate an expansion process or facilitate domain consolidation (several small, high-density domains merge into a single, large, and high-density structure).

## DISCUSSION

ChromEM maps DNA density proportionally to the measured contrast on imaging, resolving both the space-filling properties of the genome and its polymeric structure in 3D. Critically, ChromSTEM identifies a transition of chromatin from a disordered polymer (<25 nm) into higher-order structures that include packing domains and chromatin territories (*27*–*29*). By resolving the structures directly, it is evident that genome organization includes biphasic, porous packing domains (Figs. 1C and 4C) and chromatin loops (Figs. 1D and 4D) in vitro with and without RAD21 depletion. In the context of prior work, the previously observed structures of chromatin are now evident in three distinct cell line models: A549 cells (lung adenocarcinoma epithelial cells), HCT-116 cells (microsatellite unstable colonic epithelial cells), and BJ cells (immortalized fibroblasts). Although fixation alone can alter chromatin structure, prior work demonstrated that the ChromSTEM preparation protocol still maintains structural concordance with cells imaged via live-cell nanoscopy through the sample preparation stages (*29*, *38*–*40*). When combined with the similar findings observed using live-cell PWS microscopy (fig. S4) that *D* is unchanged upon RAD21 depletion, the resultant findings are concordant with chromatin organization in live cells.

**Fig. 9.**
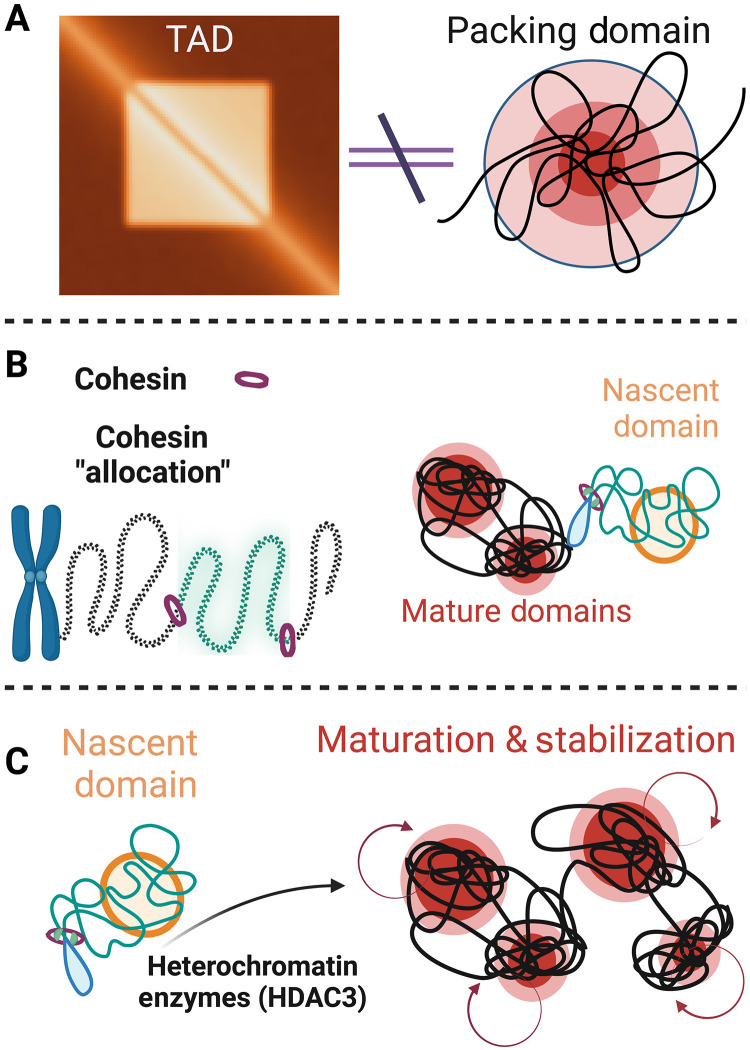
Packing domains are not the physical manifestations of TADs. (**A**) Packing domains are distinct higher-order physical structures and are not the manifestation of TADs. (**B**) Cohesin in individual cells creates genomic allocations that form into nascent domains. (**C**) The maturation of nascent domains into mature structures and their maintenance depends, at least in part, on heterochromatin enzymes such as HDAC3.

A major finding of this work is demonstrating how cohesin-mediated extrusion intersects with chromatin packing domains. While nearly all TADs and loop domains are lost in the RAD21-AID2 cell line (Fig. 3), most domains persist after depletion while retaining similar features (size, genomic content, and polymeric properties). This resilience of packing domains indicated that they are not the manifestation of TADs (Fig. 9A). Mechanistically, we uncover that RAD21 has a crucial role in domain formation, with ~50% of nascent domains lost upon 4-hour depletion. As nascent domains are defined by both their size (<80 nm) and by the packing efficiency of DNA, these findings may not be easily detectable with optical imaging modalities such as SMLM or structure illumination. We show, for example, that TADs and loop domains undergo a parallel transition. Specifically, general accessibility measured by ATAC-seq is resilient (Fig. 5) to RAD21, whereas H3K9me3 accumulates and H3K27ac decreases (Fig. 6). Together, this suggests that the cohesin’s role is to define the allocation of DNA by forming nascent domains (Fig. 9B). How these nascent domains evolve is then defined by other factors, including the heterochromatin enzyme, HDAC3 (Figs. 7 and 9C). Thus, upon RAD21 depletion, more random genomic allocations occur, resulting in the perceived increase in heterochromatin even as in situ density is unchanged. Supporting this idea is the observation using SMLM that ~94% of RAD21 is primarily positioned in interdomain space and recent work demonstrating that cohesin at TAD and loop domain anchors have similar lifetimes. While RAD21 positioning at larger domains might indicate a mechanistic function, it could alternatively be explained that simply the surface area increases with domain size. These findings collectively suggest that RAD21 does not function solely to prevent expansion.

Although this study has limitations in that it measures packing domains in a single region of one nucleus in each group, other studies have similarly showed the presence of TAD-like and domain structures after cohesin degradation (*17*, *20*–*22*, *25*). In addition, ATAC-seq and deoxyribonuclease-based methods cannot fully probe the behavior of the chromatin chain. Future work using methods with single–base pair resolution such as nucleosome occupancy and methylome sequencing (NOME-seq) could address how loops and TADs intersect with accessibility within the chromatin strand. Future research is needed to address whether previously observed TAD-like structures, chromatin nanodomains, and heterochromatic cores directly translate into packing domains. However, studies on these pending questions require the ability to identify precise genomic loci on electron microscopy while maintaining proportional mapping of stain density to DNA content without degradation of nanoscale structure (*26*).

Last, it is intriguing that Pol-II–mediated loops have similar nucleosome features to cohesin-mediated loops. Since these connections are resilient to RAD21 loss, this warrants future exploration of the role of Pol-II in packing domain function. Since prior findings show that gene transcription itself produces long-range promoter-enhancer interactions forming “loops,” the resilience of these segments could help identify the functional role of packing domains in individual cells (*25*, *41*). Supporting this is recent work showing that transcriptional inhibition via actinomycin *D* treatment results in collapse of nascent domains and swelling of the remaining domains. It is worth considering that nonenzymatic processes can have a similar impact on domain formation as forces such as ionic charge shielding or crowding could increase monomer-monomer interactions of a polymer to form long-range interactions (*22*, *25*, *42*). Alternatively, it is possible that packing domains do not emerge from one or two mechanisms but instead arise from the confluence of factors that govern both long-range interactions (loop extrusion and promoter-enhancer looping) with mechanisms that modify monomer-monomer interactions (histone methylation or acetylation and ionic shielding) like what was demonstrated in synthetic chromatin assembly (*43*, *44*). The investigation of such phenomenon in future works could expand our understanding of the molecular and physiochemical regulatory mechanisms of chromatin organization.

## MATERIALS AND METHODS

### Cell culture

HCT116 cells [American Type Culture Collection (ATCC), #CCL-247] and HCT116 RAD21-mAID-Clover CMV-OsTIR1(F74G) cells were grown in McCoy’s 5A modified medium (#16600-082, Thermo Fisher Scientific, Waltham, MA). All cell culture media were supplemented with 10% fetal bovine serum (#16000-044, Thermo Fisher Scientific, Waltham, MA) and penicillin-streptomycin (100 μg/ml; #15140-122, Thermo Fisher Scientific, Waltham, MA). Cells were maintained under recommended conditions at 37°C and 5% CO_2_. Cells were allowed at least 24 hours to readhere and recover from trypsin-induced detachment. All imaging was performed when the surface confluence of the dish was between 40 and 70%. All cells in this study were maintained between passage 5 and 20. All cells have been tested for mycoplasma contamination (ATCC, #30-1012 K) before starting experiments, and they have given negative results. To create HCT116 RAD21-mAID-Clover CMV-OsTIR1(F74G) cells, HCT116 cells (ATCC, #CCL-247) were modified with the AID system as previously described (*29*, *30*). Briefly, when the *Oryza sativa* TIR1 [OsTIR1 (F74G)] mutant is expressed in nonplant cells, it forms a Skp1–Cul1–F-box (SCF) E3 ligase complex with endogenous components. In the presence of 5-Ph-IAA, the protein of interest (RAD21) that is fused to a 7-kDa degron (mini-AID) is rapidly degraded through the ubiquitin-proteasome pathway (*29*, *30*). For HDAC3 inhibition, cells were treated with 1 μm of Rgfp966 (SML652, Sigma-Aldrich) and resuspended in DMSO for 24 hours. DMSO concentration of Rgfp966 was less than 1% (v/v).

### 5-Ph-IAA treatment

HCT116 RAD21-mAID-Clover CMV-OsTIR1(F74G) cells were plated at 50,000 cells per well of a six-well plate (Cellvis, P12-1.5H-N). To induce degradation of RAD21, 5-Ph-IAA (#HY-134653, MedChemExpress), 5-(3,4-dimethylphenyl)-indole-3-acetic acid, 5-(3-methylphenyl)-indole-3-acetic acid, and 5-(3-chlorophenyl)-indole-3-acetic acid were dissolved in DMSO to make a 500 mM stock solution and further diluted with DMSO to make a working stock solution of 1 mM immediately before the experiment. A final concentration of 1 μM 5-Ph-IAA was added to HCT116 RAD21-mAID-Clover CMV-OsTIR1(F74G) cells for 6 hours (*29*, *30*).

### Immunofluorescence imaging

HCT116 RAD21-mAID-Clover CMV-OsTIR1(F74G) cells were plated at 100,000 cells per well of a six-well glass-bottom plate (Cellvis, #P06-1.5H-N). Following auxin treatment, cells were washed twice with 1× phosphate-buffered saline (PBS) (Gibco, #10010031). Cells were fixed with 4% paraformaldehyde (Electron Microscopy Sciences, #15710) for 10 min at room temperature, followed by washing with PBS three times for 5 min each. Cells were permeabilized for 15 min using 0.2% Triton X-100 (10%) (Sigma-Aldrich, #93443) in 1× PBS, followed by another wash with 1× PBS for three times for 5 min each. Cells were blocked for 1 hour using 3% bovine serum albumin (BSA; Sigma-Aldrich, #A7906) in PBST (Tween 20 in 1× PBS) (Sigma-Aldrich, #P9416) at room temperature. The following primary antibody was added overnight at 4°C: rabbit anti-RAD21 (1:100 dilution; Abcam, #ab217678). Cells were washed with 1× PBS three times for 5 min each. Either of the following secondary antibodies was added for 1 hour at room temperature: goat anti-rabbit immunoglobulin G (IgG) H&L Alexa Fluor 568 (dilution 1:1000; Abcam, #ab175471) or goat anti-rabbit IgG H + L highly cross-adsorbed secondary antibody Alexa Fluor Plus 647 (dilution 1:500; Thermo Fisher Scientific, #A32733). Cells were washed with 1× PBS three times for 5 min each. Last, cells were stained with 4′,6-diamidino-2-phenylindole (DAPI; Thermo Fisher Scientific, #62248; diluted to 0.5 μg/ml in 1× PBS) for 10 min at room temperature. Before imaging, cells were washed with 1× PBS twice for 5 min each. The cells were imaged using the Nikon SoRa Spinning Disk confocal microscope equipped with a Hamamatsu ORCA-Fusion Digital CMOS (complementary metal-oxide semiconductor) camera. Images were collected using a 60×/1.42 numerical aperture oil-immersion objective mounted with a 2.8× magnifier. mClover was excited with a 488-nm laser, Alexa Fluor 568 was excited with a 561-nm laser, Alexa Fluor 646 was excited with a 640-nm laser, and DAPI was excited with a 405-nm laser. Imaging data were acquired by Nikon acquisition software.

### ChromSTEM-HAADF

#### Electron microscopy sample preparation

Samples were prepared as previously described (*26*–*28*). Reagents used are summarized in table S4. The cells were fixed with 2% paraformaldehyde, 2.5% glutaraldehyde [electron microscopy (EM) grade], and 2 mM CaCl_2_ in 0.1 M sodium cacodylate buffer for 30 min at room temperature and 30 min in fridge. The cells were then kept in cold temperature, if possible, for further treatments. After fixation, the cells were washed 5 × 2 min with 0.1 M sodium cacodylate buffer and blocked with 10 mM glycine, 10 mM potassium cyanide, and 0.1 M sodium cacodylate buffer for 15 min.

The cells were stained with 10 μM DRAQ5, 0.1% saponin, and 0.1 M sodium cacodylate buffer for 10 min, followed by 3 × 5 min washing with the blocking buffer. After that, the cells were photooxidized in 2.5 mM 3,3′-diaminobenzidine (EM grade) under a 100× oil objective, a 15-W xenon lamp, and a Cyanine 5 filter for 5 min. The cells were washed 5 × 2 min with 0.1 M sodium cacodylate buffer and stained with 2% osmium tetroxide, 1.5% potassium ferrocyanide, and 2 mM CaCl_2_ in 0.15 M sodium cacodylate buffer for 30 min. The cells were washed 5 × 2 min with Millipore water afterward.

The cells were then dehydrated gradually with 30, 50, 70, 85, 95%, and two times 100% ethanol. After that, the cells were incubated under room temperature with 100% ethanol and infiltrated and embedded with Durcupan resin with standard procedures. After 48 hours of resin incubation at 60°C, the resin blocks were collected for ultramicrotomy.

For ultramicrotomy, an ultramicrotome (UC7, Leica) and a 35° Diatome knife were used to section 120-nm-thick resin samples. The sections were collected on a copper slot grid (2 mm by 0.5 mm) with formvar/carbon film. Gold nanoparticles with 10-nm diameter were deposited on both surfaces of the grid afterward as fiducial markers.

#### Image collection and tomography reconstruction

Images were collected with a Hitachi HD2300 STEM microscope at 200 kV with HAADF imaging mode, at a magnification of ×50,000. Two sets of tilt series images were collected by rotating samples from −60° to +60° at a 2° step, with two roughly perpendicular rotation axes.

For postprocessing, IMOD was used to align the images. The gold nanoparticles of the collected images were removed with IMOD for another set of data without the influence of extreme values from gold nanoparticles. For each tilt series, Tomopy was used to reconstruct the volume with the penalized maximum likelihood algorithm with weighted linear and quadratic penalties. The two independent reconstructed volumes were then combined in IMOD, with gold nanoparticles as the matching model and repeated on the data without nanoparticles.

After reconstruction, the top and the bottom 0.1% pixel values were capped to remove extreme values. The pixel values are then scaled between 0 and 1 for analysis.

#### Chromatin domain identification and analysis

Chromatin domains were identified and analyzed following the approach previously mentioned (*27*, *28*). For identification of domains, a Gaussian filter with radius = 5 pixels was used followed by contrast-limited adaptive histogram equalization (CLAHE) contrast enhancement on the 2D projection of the 3D tomogram using ImageJ. Chromatin domain centers were identified as local maxima with prominence = 1.5 × SD of pixel values.

For domain properties, an 11 × 11 pixels window is applied to each domain and sampled for each domain. Packing scaling analysis is done by measuring the total intensity of the chromatin that radially expands from the center pixel picked and weighted by the intensity of the center pixel. The linear region of the packing scaling behavior is identified by MATLAB “ischange” function. The domain size is measured as the point at which the packing scaling behavior deviates from the linear fits with 5% difference or when local packing scaling exponent *D* reaches 3. CVC of a domain is measured with the average value within the domain on a binarized image with the Otsu binarization algorithm after CLAHE contrast enhancement in ImageJ. The packing efficiency, *A*, is calculated as previously described from the relationship described previously (*27*, *28*). Briefly, *I* = A*(*R*_f_ */R*_min_)*^D^*, where *I* is the average chromatin intensity of a domain, *R*_f_ is the size of the domain, *R*_min_ = 10 nm is the smallest unit of random chromatin polymer chain, and *D* is the packing scaling exponent of the domain packing behavior. Loop images and videos were generated from the produced tomograms and then underwent background subtraction with an applied local filter for visualization.

### Micrococcal nuclease chromatin conformation capture data processing and analysis

Processed Micro-C files were obtained from publicly available data through the ENCODE Consortium (table S1) (*31*–*33*). TAD and loop domains were obtained from the reported bedpe files (table S1). Compartment eigenvector analysis and Pearson correlation analysis were generated using Juicer’s eigenvector and Pearsons scripts, respectively, or using built-in functions in GENOVA. Before analysis, Hi-C contacts were dumped using Juicer’s straw (https://github.com/aidenlab/straw). Contacts were converted to coolers and normalized using Cooler (https://github.com/open2c/cooler) at 5- and 100-kb resolution. Aggregate TAD analysis and aggregate peak analysis were generated on these contact maps using GENOVA (https://github.com/robinweide/GENOVA). Visualization of contact loci was also done using GENOVA. All other analyses were custom-generated in R. All code used in this publication are available on GitHub at https://github.com/BackmanLab.

### Assay for ATAC-seq, low input, Mint-ChIP-seq, and Chia-PET sequencing

Processed ATAC-seq bed files, multiplexed chromatin immunoprecipitation sequencing (Mint-ChIP-seq), and Chia-PET files were obtained from publicly available data through the ENCODE Consortium (tables S2 and S3) (*31*–*33*). Analysis of global accessibility was performed by analyzing the per-chromosome number of accessible loci previously identified with a *q* value threshold greater than 1. The location of each peak was approximated by the average position reported between the 3′ and 5′ locations. Analysis of accessibility within TADs and loop domains was performed by using the indices reported above (table S1). For loop domains, the mean 3′ and 5′ ends for each anchor locus were calculated and used for subsequent analysis of accessibility. Within each individual TAD or loop location, the relative accessibility was calculated by measuring the change in the number of peaks within each TAD or loop domain after RAD21 depletion. For analysis of histone marker distributions, the number of loci that were identified on (i) each chromosome or (ii) within loop/TAD boundaries was then calculated before and after RAD21 depletion in HCT-116 cells. With respect to Chia-PET analysis, the frequency of loop events of CTCF and RNA polymerase II subunit A (POLR2A) was calculated and binned by the frequency of events observed within the population. Analysis was restricted to loops with at least five observed events in each group.

### Single-molecule localization microscopy

#### Multicolor SMLM sample preparation

The primary antibody rabbit anti-RAD21 (Abcam, #ab217678) was aliquoted and stored at −20°C. The secondary antibody goat anti-rabbit Alexa Fluor 488 (Thermo Fisher Scientific) was stored at 4°C. EdU staining kit components (Thermo Fisher Scientific) were stored per the manufacturer’s protocol after resuspension of reagent components.

Two-color SMLM sample preparation is done via two sequential staining processes for the two targets.

1) Cells were plated on no. 1 borosilicate-bottom eight-well Lab-Tek Chambered cover glass at a seeding density of 12.5 to 25,000. After 48 hours, the cells were incubated with EdU overnight to undergo a full cell cycle and then underwent fixation for 10 min at room temperature with a fixation buffer composed of 4% paraformaldehyde in PBS. Samples were then washed in PBS for 5 min three times. Secondary staining via click reaction chemistry was performed as described in the manufacturer’s protocol.

2) Permeabilization was then done with blocking buffer composed of (3% BSA and 0.5% Triton X-100 in PBS) for 1 hour, and then samples were immediately incubated with rabbit anti-Rad21 (Abcam) in blocking buffer for 1 to 2 hours at room temperature on a shaker. Samples were then washed three times with a washing buffer composed of 0.2% BSA and 0.1% Triton X-100 in PBS.

3) Samples were then incubated with the corresponding goat antibody–dye conjugates and anti-rabbit AF488 (Thermo Fisher Scientific) for 40 to 60 min at room temperature on the shaker. After incubation, samples were washed two times in PBS for 5 min on a shaker, and then samples were then imaged. Ten thousand frames were acquired for each respective channel after depletion for each cell.

#### Single-molecule localization data analysis

Acquired data were first processed using the ThunderSTORM ImageJ (*45*) plugin to generate the reconstructed images for visualization via the average shifted histogram method, as well as the localization datasets. Each localization dataset was corrected for drift. Localization coordinates (*x* and *y*) were then used in a Python point-cloud data analysis algorithm, which used the scikit-learn density-based spatial clustering of applications with noise (DBSCAN) method (parameter choice: 50-nm maximum distance between points and a minimum of three points per cluster) to cluster the DNA density localizations. Cluster size was determined by the area of the convex hull fit of the clustered marks and then normalized relative to a circular cluster with radius of 80 nm. Clusters were demarcated into two groups: large cluster (>80 nm) and small cluster (20 to 80 nm). Domains calculated to be >800 nm were excluded as they likely represented the nuclear boundary. Sample density was measured by counting the number of corresponding emission markers in a concentric convex hull from the identified cluster center to extend to 60 nm. RAD21 association was determined by measuring the number of molecules within the boundary of the domain plus the bounding area of 60 nm. Data shown are for concatenation of *n* = 12 cells from two technical replicates.

### Live-cell PWS microscopy

For live-cell measurements, cells were imaged and maintained under physiological conditions (5% CO_2_ and 37°C) using a stage-top incubator (In Vivo Scientific, Salem, SC; Stage Top Systems). The PWS optical instrument was built on a commercial inverted microscope (Leica, Buffalo Grove, IL, DM IRB) supplemented with a Hamamatsu Image EM charge-coupled device camera C9100-13 coupled to a liquid crystal tunable filter (Cambridge Research and Instrumentation Woburn, MA) for hyperspectral imaging. Spectrally resolved images of live cells were collected between 500 and 700 nm with a 2-nm step size (*28*, *37*). Broadband illumination was provided by an Xcite-120 light-emitting diode lamp (Excelitas, Waltham, MA). PWS is a high-throughput, label-free technique that measures the spectral SD (Σ) of internal optical scattering originating from chromatin (*37*). Variations in the refractive index distribution, Σ, are characterized by a mass density autocorrelation function to calculate chromatin packing, scaling *D* (*28*). Changes in *D* resulting from each condition are quantified by averaging cells, taken across three technical replicates. Population *D* is calculated by first averaging *D* values from PWS measurements within each cell nucleus and then averaging these measurements over the entire cell population for each treatment condition. Since *D* is uncorrelated from cell to cell except for direct progeny, statistical comparison is performed across the whole population instead of on the technical replicates (*28*).

### Western blot analysis

Total cellular protein from HCT116 RAD21-mAID-Clover CMV-OsTIR1(F74G) cells was extracted using the Western-Ready Rapid Protein Extraction Buffer (BioLegend, #426305) following the manufacturer’s protocol. Cell lysates were quantified with a standard Bradford assay using the Pierce Bradford Plus Protein Assay Reagent (Thermo Fisher Scientific, #23238) and Pierce BSA Standard Pre-Diluted Set (Thermo Fisher Scientific, #23208). Heat-denatured protein samples were resolved on a 4 to 12% Bolt Bis-Tris Plus Mini Gel (Invitrogen, #NW04125BOX) and transferred to a polyvinylidene difluoride membrane using the iBlot 2 Gel Transfer Device (Invitrogen, #IB21001) (20 V for 7 min). Antibodies were bound to the membrane using the iBind Flex Western System (Invitrogen, #SLF2000) and associated reagents. Whole-cell lysates were blotted against the following primary antibodies: anti-RAD21 (dilution 1:200; Abcam, #ab217678) and anti–glyceraldehyde phosphate dehydrogenase (GAPDH; dilution 1:1000; Sigma-Aldrich, #G9545). The following secondary antibody was used: goat anti-rabbit IgG (heavy chain), superclonal recombinant secondary antibody, horseradish peroxidase (dilution 1:4000; Invitrogen, #A27036). To develop blots for protein detection, the SuperSignal West Pico PLUS Chemiluminescent Substrate (Thermo Fisher Scientific, #34580) was used. To quantify the Western blot bands, we used the iBright CL1500 Imaging System (Invitrogen, #A44240) and iBright Analysis Software to define bands as regions of interest. Samples were loaded in duplicate on the same gel. The blot was then sectioned in half after transfer to facilitate detection of GAPDH and RAD21.

Samples were loaded in duplicate on the same gel. The blot was then sectioned in half after transfer to facilitate detection of either RAD21 or GAPDH.

### Utilization of artificial intelligence tools

Artificial intelligence (AI) tools were used within this manuscript as follows. Commands were given to ChatGPT 4.0 to assess the logic structure of written portions and redrafted if the wrong conclusions were identified from the provided passages. For example, the following prompt of “please summarize what the following points are from this discussion” with paragraphs from that section. Final proof editing and revisions of the manuscript were performed by the authors. Furthermore, AI tools were not used for the direct writing of the manuscript. For assistance with visualization of data, ChatGPT 4.0 was prompted to generate a code to produce error bars within the plot as with the following commands: “How do I generate listplot in mathematica with error bars” which produced an obsolete code. This was addressed with further prompting “Errorbarplots are obsolete, is there a new way.” The produced code was then modified with the relevant data and error points. The visualization was likewise modified for clarity.

### Statistical analysis

Statistical analysis was performed using GraphPad Prism 10.1.1, Microsoft Excel, and Mathematica. Pairwise comparisons were calculated on datasets consisting of, at a minimum, biologically independent duplicate samples using two-tailed unpaired *t* test. The type of statistical test is specified in each case. A *P* value of < 0.05 was considered significant. Sample number (# of nuclei, *n*) packing domains and the type of statistical test used are indicated in figure legends. Corrections for multiple comparisons on the same data were considered, and Bonferroni correction was applied as indicated where appropriate.
