# Supplementary material for: Mature chromatin packing domains persist after RAD21 depletion in 3D
Source: Sci Adv. 2025 Jan 24;11(4):eadp0855. doi: 10.1126/sciadv.adp0855 (PMC11759041; doi:10.1126/sciadv.adp0855)
Supplement: Supplementary file 1 — Figs. S1 to S7 Tables S1 to S4 Legends for movies S1 to S4 [file sciadv.adp0855_sm.pdf]

Science Advances
AAAS

Supplementary Materials for

Mature chromatin packing domains persist after RAD21 depletion in 3D

Wing Shun Li *et al.*

Corresponding author: Vadim Backman, v-backman@northwestern.edu

Sci. Adv. **11**, eadp0855 (2025)
DOI: 10.1126/sciadv.adp0855

The PDF file includes:

Figs. S1 to S7
Tables S1 to S4
Legends for movies S1 to S4

Other Supplementary Material for this manuscript includes the following:

Movies S1 to S4

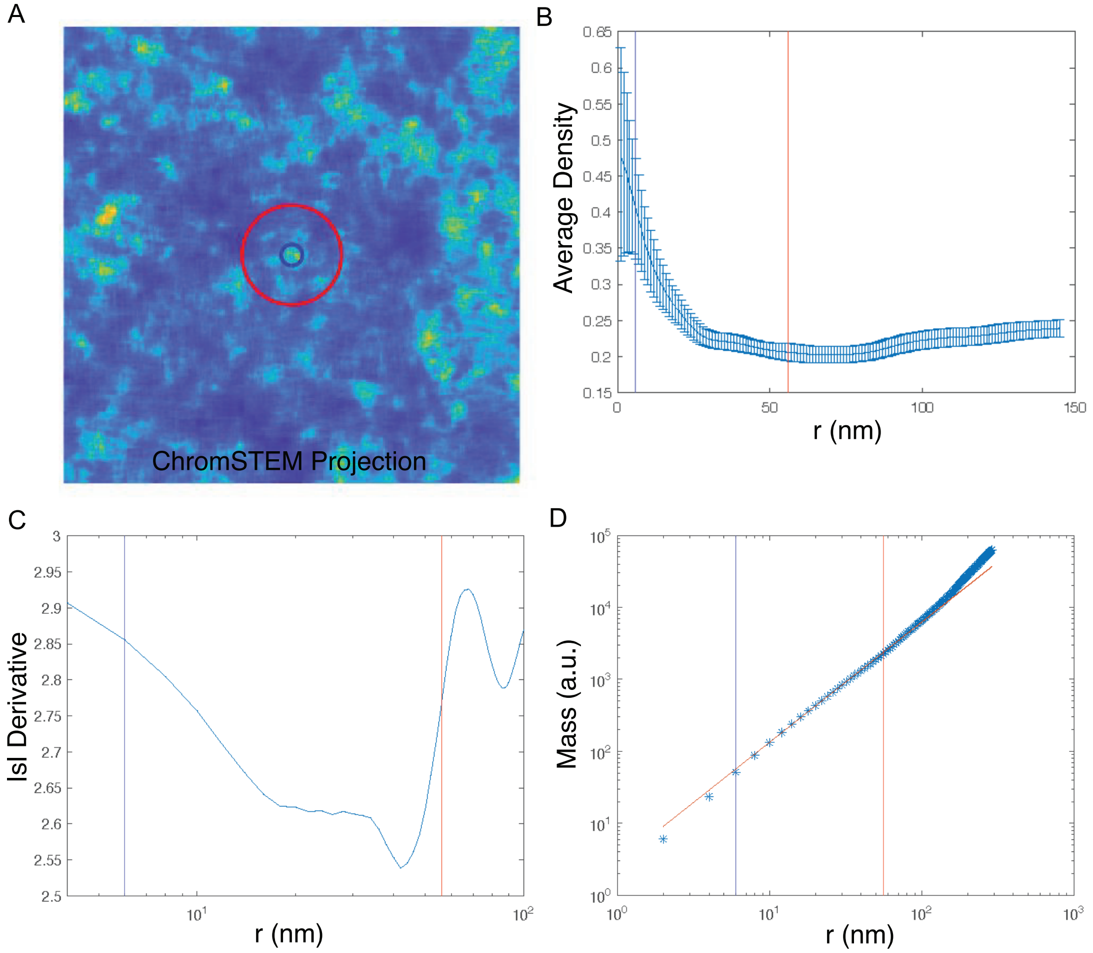

Fig. S1. Identification of packing domain boundaries. A) Representative packing domain center with surrounding calculated radius. **B)** Analysis of the radial distribution of mass from the center of the domain to identify the boundary. The boundary by this method is defined by the point where the mass is comparable to the average mass throughout the tomogram. **C)** Identification of domain boundary by calculating the point where the first derivative of the mass scaling equals 3. **D)** Analysis of domain radius by the radial density distribution function. The boundary by this method is defined by the radius where the mass deviates from power-law scaling (deviation from the log-log fit). The minimum radius from these three methods is then defined as the boundary of the packing domain.

A

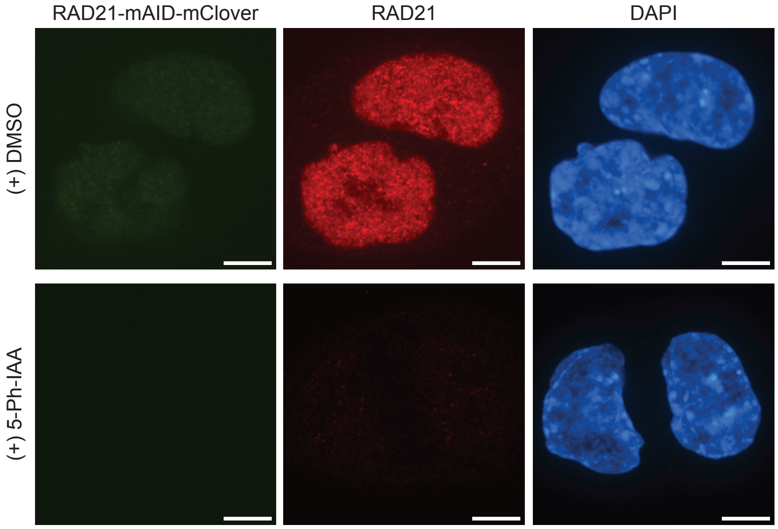

Fig. S2. Representative images of DMSO controls and RAD21(-) at 6 hours. Representative fluorescence image of control cells vs RAD21 depleted cells at 6 hours. First column represents mClover signal, second column represents immunofluorescence signal from RAD21 antibody staining, third column represents DAPI nuclear counter staining. Scale bar represents 5 microns.

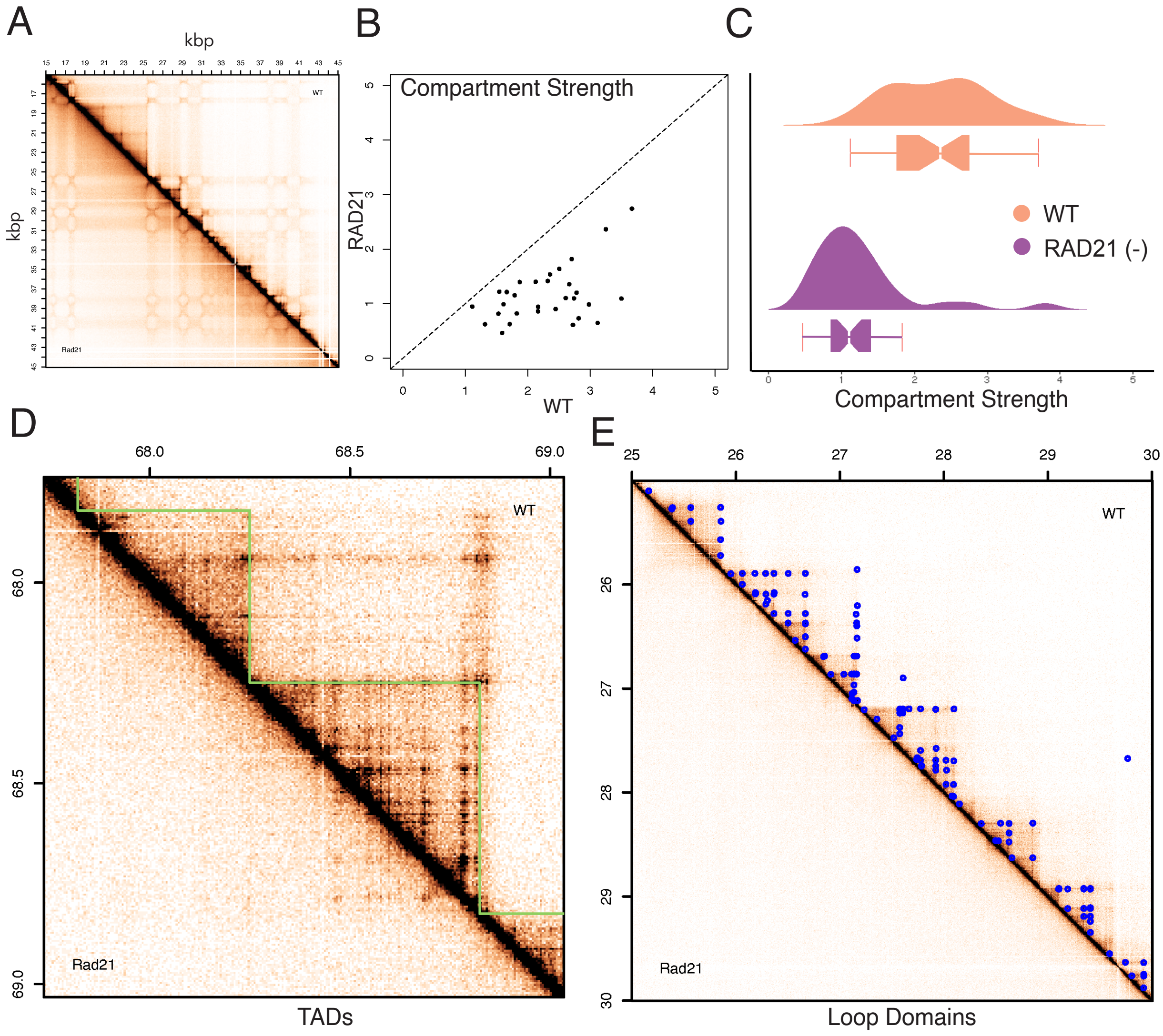

Fig. S3. Extended analysis of DMSO control and RAD21(-) Micro-C features. A) Representative change in compartments upon RAD21 depletion. **B)** Alterations in compartment strength per chromosome. **C)** Genome-wide transformation of compartment strength. D) Representative TAD loci before and after RAD21 depletion. **E)** Representative loop domains before and after RAD21 depletion

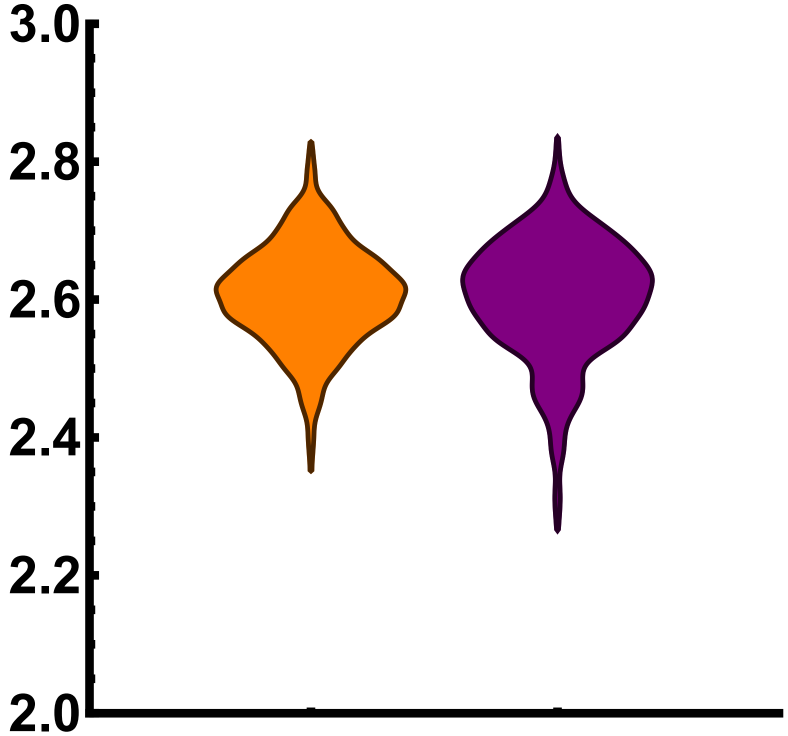

Fig. S4 Live-cell Partial Wave Spectroscopic imaging of HCT116 cells, DMSO control, and RAD21(-) cells. Comparison of chromatin organization in DMSO treated control cells (orange, n=916 cells from 3 replicates; mean *D* of 2.61 with a standard deviation of 0.072) in comparison to 5-Ph-IAA treated cells at 4 hours (purple, n = 922 from 3 replicates, mean *D* of 2.60 with standard deviation of 0.086). Two-tailed, unpaired T-test with p-value of 0.104.

Uncropped Western Blots

1st Replicate   2nd Replicate

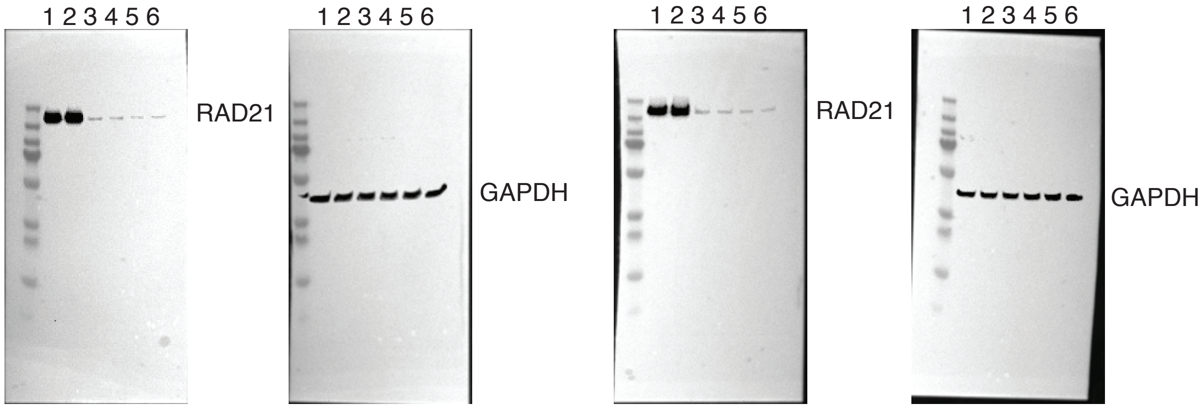

NOTES:

- 6 µg loaded per well of HCT116 RAD21-mAID-Clover CMV-OsTIR1 (F74G) lysate
- Order of Lanes:
  1. Untreated
  2. 6 hour DMSO
  3. 1 hour 1µM 5-Ph-IAA
  4. 2 hour 1µM 5-Ph-IAA
  5. 4 hour 1µM 5-Ph-IAA
  6. 6-hour 1µM 5-Ph-IAA
- RAD21 bands at ~135 kDa
- GAPDH bands at ~37 kDa
- Ladder: PageRuler Plus Prestained Protein Ladder (Thermo Fisher Scientific, Cat: 26619)

Fig. S5 Western Blot analysis of RAD21 depletion over time after 5-Ph-IAA treatment. Rapid depletion of RAD21 is achieved within 1 hour of exposure to 1 micromolar of 5-Ph-IAA in HCT RAD21-mAID-Clover CMV-OsTIR1(F74G) cells.

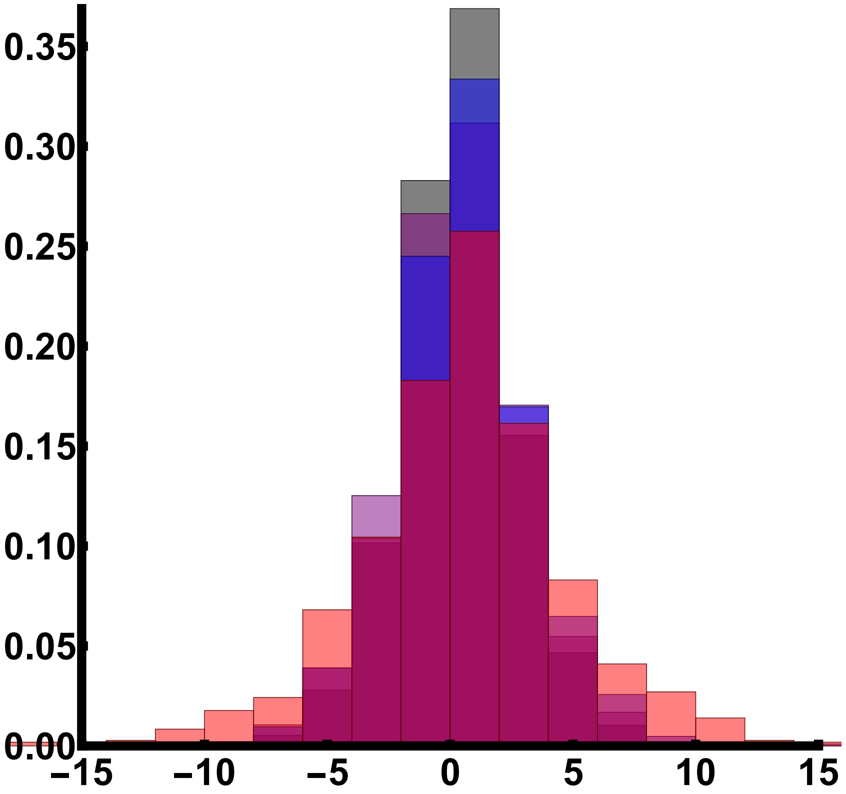

Fig. S6 Analysis of accessibility within TADs as a function of size. Analysis of the change in number of ATAC-Seq loci within TAD coordinates before and after RAD21 depletion showing no change in accessibility in TAD regions upon their loss independent of their size. Median change is zero in all groups. Largest quartile (>320Kbp, Red; IQ -2 to +2). Third quartile (between 130 and 320Kbp, Blue; IQ -2 to +2). Second quartile (between 90Kbp and 130Kbp, Purple; IQ -2 to +1). Smallest quartile (<90Kbp, black; IQ -1 to +1). Y-axis (probability). X-axis (change in loci ATAC-Seq peaks after RAD21 depletion).

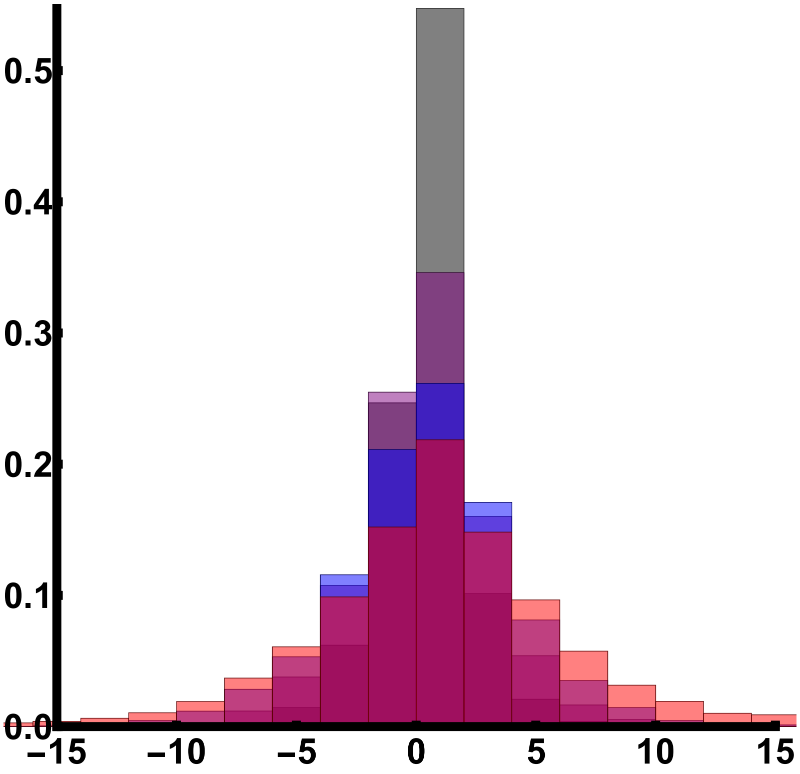

Fig. S7 Analysis of accessibility within loop domains as a function of size. Analysis of the change in number of ATAC-Seq loci within loop domain coordinates before and after RAD21 depletion showing no change in accessibility in loop domain regions upon their loss independent of their size. Median change is zero in all groups. Largest quartile (>360Kbp, Red; IQ -2 to +3). Third quartile (between 168 and 360Kbp, Blue; IQ -2 to +2). Second quartile (between 70Kbp and 168Kbp, Purple; IQ -2 to +1). Smallest quartile (<70Kbp, black; IQ -1 to +1). Y-axis (probability). X-axis (change in loci ATAC-Seq peaks after RAD21 depletion).

Table S1. Micro-C ENCODE Consortium Files Utilized

Experiment	File	Method	Source	Type
**ENCSR958BEA**	ENCFF794OIH	Micro-C	Genetically modified HCT-116 cell lines with CRISPR inserting O. sativa LOC4335696. Untreated.	Bedpe file; loop domains
**ENCSR958BEA**	ENCFF334LVK	Micro-C	Genetically modified HCT-116 cell lines with CRISPR inserting O. sativa LOC4335696. Untreated.	Bedpe file; contact domains
**ENCSR958BEA**	ENCFF528XGK	Micro-C	Genetically modified HCT-116 cell lines with CRISPR inserting O. sativa LOC4335696. Untreated.	.hic file; contact matrix
**ENCSR087JOM**	ENCFF630HHH	Micro-C	Genetically modified HCT-116 cell lines with CRISPR inserting O. sativa LOC4335696 treated with 5-Ph-IAA for 6 hours for RAD21 depletion	Bedpe file; loop domains
**ENCSR087JOM**	ENCFF307DPS	Micro-C	Genetically modified HCT-116 cell lines with CRISPR inserting O. sativa LOC4335696 treated with 5-Ph-IAA for 6 hours for RAD21 depletion	Bedpe file; contact domains
**ENCSR087JOM**	ENCFF317OIA	Micro-C	Genetically modified HCT-116 cell lines with CRISPR inserting O. sativa LOC4335696 treated with 5-Ph-IAA for 6 hours for RAD21 depletion	.hic file; contact matrix

Table S2. ATAC-Seq ENCODE Consortium Files Utilized

Experiment	Bed File	Method	Source
**ENCSR135OML**	ENCFF460JUY	ATAC-Seq	Genetically modified HCT-116 cell lines with CRISPR inserting O. sativa LOC4335696 treated with 5-Ph-IAA for 6 hours for RAD21 depletion
**ENCSR389WJO**	ENCFF107INQ	ATAC-Seq	Genetically modified HCT-116 cell lines with CRISPR inserting O. sativa LOC4335696

Table S3. ChIP-Seq and Chia-PET ENCODE Consortium Files Utilized

Experiment	Bed/Bedpe File	Method	Source
**ENCSR871TIY**	ENCFF387HBG	Mint-ChIP-Seq	H3K27ac. Genetically modified HCT-116 cell lines with CRISPR inserting O. sativa LOC4335696 treated with 5-Ph-IAA for 6 hours for RAD21 depletion
**ENCSR808WHQ**	ENCFF391JYF	Mint-ChIP-Seq	H3K9me3. Genetically modified HCT-116 cell lines with CRISPR inserting O. sativa LOC4335696 treated with 5-Ph-IAA for 6 hours for RAD21 depletion
**ENCSR323XBZ**	ENCFF134CXL	Mint-ChIP-Seq	H3K27ac. Genetically modified HCT-116 cell lines with CRISPR inserting O. sativa LOC4335696 untreated
**ENCSR412FVA**	ENCFF394DVE	Mint-ChIP-Seq	H3K9me3. Genetically modified HCT-116 cell lines with CRISPR inserting O. sativa LOC4335696 untreated
**ENCSR771PKY**	ENCFF718SMU	Chia-PET	POLR2A. Genetically modified HCT-116 cell lines with CRISPR inserting O. sativa LOC4335696 treated with 5-Ph-IAA for 6 hours for RAD21 depletion

**ENCSR281XBJ**	ENCFF751AJI	Chia-PET	CTCF. Genetically modified HCT-116 cell lines with CRISPR inserting O. sativa LOC4335696 treated with 5-Ph-IAA for 6 hours for RAD21 depletion
**ENCSR353XNY**	ENCFF511YYL	Chia-PET	POLR2A. Genetically modified HCT-116 cell lines with CRISPR inserting O. sativa LOC4335696 untreated
**ENCSR035PVZ**	ENCFF322FOT	Chia-PET	POLR2A. HCT-116 cell lines.
**ENCSR278IZK**	ENCFF431BUN	Chia-PET	CTCF. HCT-116 cell lines.

Table S4. Reagents used in ChromSTEM sample preparation

Reagent	Formula
Washing solution	Hank's balanced salt solution without calcium and magnesium
Fixation solution	2.5% EM grade glutaraldehyde<br>2% paraformaldehyde<br>2 mM $CaCl_2$<br>0.1 M sodium cacodylate buffer, pH = 7.4
Blocking solution	10 mM glycine<br>10 mM potassium cyanide<br>0.1 M sodium cacodylate buffer, pH = 7.4
DNA staining solution	10 μM DRAQ5<br>0.1% SAPONIN<br>0.1 M sodium cacodylate buffer, pH = 7.4
Bathing solution	2.5 mM 3,3'- diaminobenzidine tetrahydrochloride (DAB)<br>0.1 M sodium cacodylate buffer, pH = 7.4
Reduced osmium staining solution	2% osmium tetroxide<br>1.5% potassium ferrocyanide<br>2 mM $CaCl_2$<br>0.15 M sodium cacodylate buffer, pH = 7.4
Durcupan ™ resin mixture 1	10 mL Durcupan ™ ACM single component A, M, epoxy resin<br>10 mL Durcupan ™ ACM single component B, hardener 964<br>0.15 mL Durcupan ™ ACM single component D
Durcupan ™ resin mixture 2	10 mL Durcupan ™ ACM single component A, M, epoxy resin<br>10 mL Durcupan ™ ACM single component B, hardener 964<br>0.2 mL Durcupan ™ ACM, single component C, accelerator 960<br>0.15 mL Durcupan ™ ACM single component D
1:1 infiltration mixture	10 mL 100% ethanol<br>10 mL Durcupan ™ resin mixture 1
2:1 infiltration mixture	5 mL 100% ethanol<br>10 mL Durcupan ™ resin mixture 1

Movie S1. **3D ChromSTEM-HAADF reconstruction of a loop in control HCT-116 cells.** Representative 3D tomogram of a loop domain within control HCT-116 cells demonstrating long range interactions. Raw data was modified with background subtraction and gaussian filter application for visualization.

Movie S2. **3D ChromSTEM-HAADF reconstruction of a packing domain in control HCT-116 cells.** Reconstructed tomogram of a chromatin packing domain within control HCT-116 cells.

Movie S3. **3D ChromSTEM-HAADF reconstruction of a loop in RAD21(-) HCT-116 cells** Representative 3D tomogram of a loop domain within 5-Ph-IAA treated HCT-116 cells demonstrating long range interactions. Raw data was modified with background subtraction and gaussian filter application for visualization.

Movie S4. **3D ChromSTEM-HAADF reconstruction of a packing domain in RAD21(-) HCT-116 cells.** Representative 3D tomogram of a loop domain within 5-Ph-IAA treated HCT-116 cells after 4 hours of treatment.
